# An anti-biofilm cyclic peptide targets a secreted aminopeptidase from *P. aeruginosa*

**DOI:** 10.1038/s41589-023-01373-8

**Published:** 2023-06-29

**Authors:** Christopher John Harding, Marcus Bischoff, Megan Bergkessel, Clarissa Melo Czekster

**Affiliations:** 1 Biomedical Sciences Research Complex, School of Biology, University of St Andrews, St Andrews, UK; 2 Centre of Biophotonics, University of St Andrews, St Andrews, UK; 3 School of Life Sciences, University of Dundee, Dundee, UK

**Keywords:** Proteases, X-ray crystallography, Peptides, Microbiology

## Abstract

*Pseudomonas aeruginosa* is an opportunistic pathogen that causes serious illness, especially in immunocompromised individuals. *P. aeruginosa* forms biofilms that contribute to growth and persistence in a wide range of environments. Here we investigated the aminopeptidase, *P. aeruginosa* aminopeptidase (PaAP) from *P. aeruginosa*, which is highly abundant in the biofilm matrix. PaAP is associated with biofilm development and contributes to nutrient recycling. We confirmed that post-translational processing was required for activation and PaAP is a promiscuous aminopeptidase acting on unstructured regions of peptides and proteins. Crystal structures of wild-type enzymes and variants revealed the mechanism of autoinhibition, whereby the C-terminal propeptide locks the protease-associated domain and the catalytic peptidase domain into a self-inhibited conformation. Inspired by this, we designed a highly potent small cyclic-peptide inhibitor that recapitulates the deleterious phenotype observed with a PaAP deletion variant in biofilm assays and present a path toward targeting secreted proteins in a biofilm context.

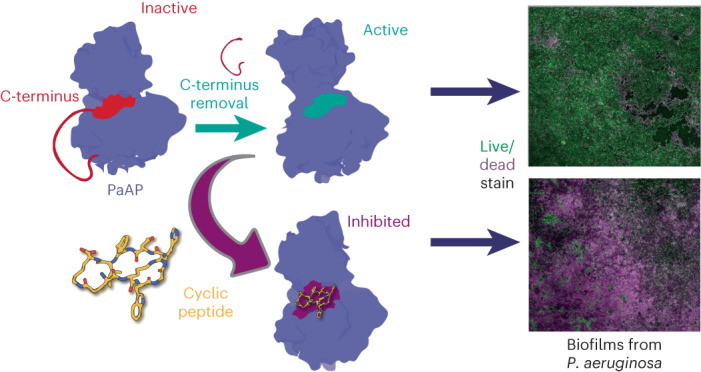

## Abstract (web summary)

The secreted aminopeptidase *Pseudomonas* *aeruginosa* aminopeptidase (PaAP) is required for nutrient recycling in biofilms. Using the information from protein structure and kinetics, a potent cyclic peptide inhibitor for PaAP was designed that killed cells in late-stage biofilms.

## Main

*Pseudomonas aeruginosa* is an environmental bacterium that colonizes a wide range of aquatic and terrestrial habitats. It is a well-known opportunistic human pathogen causing both acute and chronic diseases. Moreover, it is one of the leading causes of chronic infection in cystic fibrosis where its ability to adapt and thrive in different environments makes it incredibly difficult to treat^1,2^. The survival strategies of *P. aeruginosa* include its ability to form complex biofilms and secrete numerous virulence factors^3–6^. Secreted virulence factors specifically enhance pathogen survival in a host context and, for *P. aeruginosa*, encompass extracellular polymeric substances, which include the exopolysaccharides alginate, Pel and Psl; outer membrane vesicles (OMV)^7,8^; siderophores, such as pyoverdine and pyochelin; toxins such as exotoxin A and pyocyanin; and lytic enzymes, such as proteases and nucleases. Many of these secreted factors cause cell and tissue damage, interfere with host defenses and promote bacterial growth and proliferation^9^.

Secreted proteases are considered major virulence factors of *P. aeruginosa*. These include an alkaline protease, elastases (LasA and LasB) and a lysin-specific endopeptidase (protease IV, also referred to as LysC)^9,10^. LasB degrades collagens, causing severe tissue damage^11^. It also interferes with host defenses by degrading several innate immune components^12,13^. Alkaline protease degrades many host proteins, including fibronectin and laminin, playing an important role in invasion and tissue necrosis. It is an important protein to escape bacterial defenses by degrading complement proteins and cytokines^14^. Protease IV is mainly associated with virulence in corneal infections^15,16^. It is involved in tissue damage and invasion by degrading fibrinogen.

Recently, attention has been directed to exploring other secreted proteases that may contribute to *P. aeruginosa* success and complement the activity of the endopeptidases described above. The secreted exopeptidase, *P.* *aeruginosa* aminopeptidase (PaAP; *pepB*, PA14_26020), is one of the most abundant proteins in the biofilm matrix^17^. Interestingly, PaAP is also the major protein associated with *P. aeruginosa* OMVs. These spherical bilayered phospholipids encapsulate proteins, lipopolysaccharides, and DNA and have been implicated in important biological functions such as virulence and nutrient acquisition^7^.

PaAP is annotated as an aminopeptidase, which removes amino acids from the N-termini of peptides, with a preference for leucine^18^. It is linked to biofilm development, where it is suggested to have a role in nutrient recycling, leading to its characterization as a ‘public good’ enzyme (an enzyme that benefits both the producing and nonproducing micro-organisms)^19^. Its expression is dependent on the stress sigma factor RpoS and is affected by quorum-sensing (QS) systems^20–22^. Moreover, PaAP requires post-translational processing to become an active enzyme. It is secreted through the type II secretion system (also regulated by the QS system) into the extracellular matrix^4^. Once secreted, PaAP undergoes proteolytic processing by other extracellular matrix proteases, such as truncation of the short C-terminal propeptide region by LysC^23^. Before our work, a precise molecular role for C-terminal truncation was absent.

Here we investigated the structure and function of PaAP, its catalytic and regulatory mechanism and its importance during biofilm development. We determined the elegant regulatory mechanism whereby the C-terminal propeptide region of PaAP binds in a groove between the protease and protease-associated (PA) domain, blocking access to the active site pocket. Moreover, we provide evidence that the PA domain functions as a regulator of enzyme activity. Finally, aided by structural insights, we rationally designed a specific and potent peptide inhibitor of the peptidase, which impacts late-stage biofilms as well as planktonic growth, where aminopeptidase activity is required. Our work reveals crucial insights into the secreted protease PaAP and provides a new strategy for the development of anti-biofilm peptides targeting extracellular factors.

## Results

### Structure of PaAP reveals autoinhibition by C-terminus

**Fig. 1:**
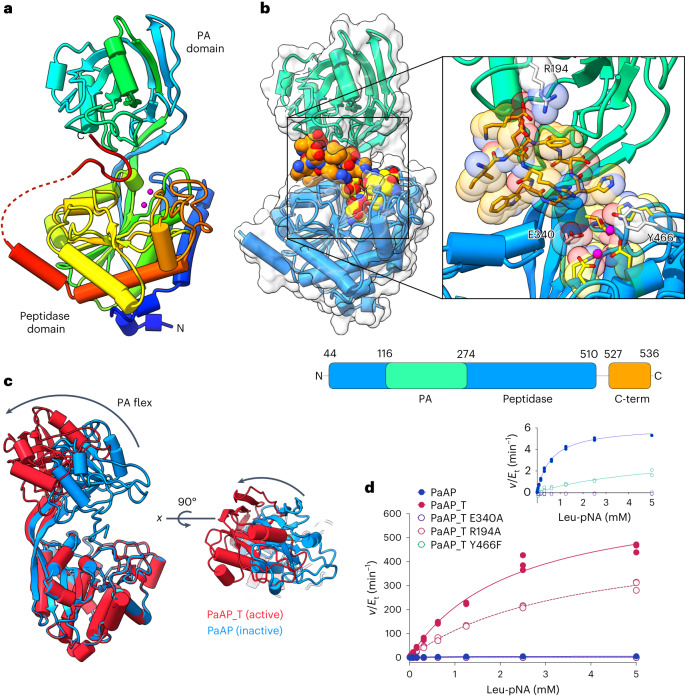
Mechanism of autoinhibition. **a**, The crystal structure of WT PaAP. The secondary structure is colored in rainbow, blue N-terminus through to red C-terminus. Pink spheres are zinc ions that indicate the location of the active site. A short region of disorder between the C-terminus and the peptidase domain is shown by a dashed red line. **b**, Structure of PaAP with surface displayed demonstrates the closed inactive conformation. PA domain is shown in turquoise and peptidase domain in blue. The active site (yellow residues) is occluded by the C-terminus (orange residues), which binds in a groove between the PA and peptidase domains. **c**, The structure of WT PaAP (blue) in comparison to the PaAP_T (red), which lacks the C-terminus, illustrates the conformational change of the PA domain, which allows access to the active site. A Supplementary Video depicts this conformational change. **d**, The activity of WT PaAP versus PaAP_T (solid circles) and PaAP_T variants (white circles). PaAP shows higher activity upon removal of the inhibitory C-terminus. The inset panel on the left shows zoomed-in view of *v*/*E*_t_ values to show the reduced activity of other PaAP forms. Experiments were performed in triplicate. Source data

We solved the structure of PaAP in multiple forms, which revealed an interesting self-inhibitory mechanism involving the PA domain and the propeptide C-terminus (Fig. 1a). The structure of full-length PaAP was solved to 1.4 Å, and traceable between residues 44 to 536 with a 16 amino acid region (QKAQSRSLQMQKSASQ) near the C-terminus, which lacked density. However, the final ten amino acids (IERWGHDFIK) of the C-terminus could be unambiguously traced (Fig. 1b). The structure of PaAP is similar to other unpublished aminopeptidases that have been uploaded onto the Protein Data Bank (PDB: 2EK8 and 6HC6; Supplementary Fig. 1a). PaAP contains an M28 Zn peptidase domain (residues 44–116 and 274–510) and a PA domain (residues 117–273; Fig. 1a). The aminopeptidase domain is composed of an eight-stranded β-sheet surrounded by helices. The smaller mixed α/β PA domain is attached to the peptidase domain via an extended β-strand. There are three disulfide bonds—two in the peptidase domain, toward the N- and C- termini and one in the PA domain.

The active site contains two zinc ions; Zn1 is coordinated by D306, H296 and D369, while Zn2 is coordinated by E341, H467 and D306. Coordinated between both Zn ions is a water molecule, whose activation to OH^−^ is crucial for catalysis. Residues, E340 and Y466, complete the active site (Supplementary Fig. 2).

Interestingly, in the structure of PaAP, following a short region of disorder, the C-terminus (IERWGHDFIK, residues 527–536) binds in the cleft between the PA and peptidase domain (Fig. 1b). This short propeptide region is structured, folding with a hairpin turn. A short (1-2aa) β-sheet of four strands runs through the C-terminus and PA domain (Supplementary Figs. 3 and 4). The carboxy terminus interacts with the side chain of R194. Side chains of H532 and D533 of the β-hairpin coordinate a water molecule and are positioned directly above the active site pocket. The location of the C-terminus appears to block access to the active site residues.

We solved the structure of the truncated enzyme form lacking the final C-terminal residues, PaAP_T, to 2.35 Å, which revealed a large conformational change. Superimposition of the peptidase domains of full-length PaAP and PaAP_T demonstrates the different orientations adopted by the PA domain due to the removal of the C-terminus. The PA domain undergoes an ~40° rotational transition in comparison to the full-length PaAP (20 Å translation at its furthest point). The conformational change increases the opening between the PA and peptidase domain, causing the active site to be more accessible (Fig. 1c). The rotational swing occurs from the base of the two extended β strands that project from the peptidase domain (starting at P161 and E272). The PA domain was also captured in an alternative conformation in the truncated construct, PaAP_T_E340A(trunc)_ (Supplementary Fig. 5a). These observations imply that the PA domain becomes free to rotate when not locked into an inactive conformation by the C-terminus interaction.

Interestingly, the modified C-terminus (thrombin cleavage site, LVPR) is fully traceable in the PaAP_T structure and binds to the PA domain of an adjacent protomer, where the C-terminus interacts directly with R189A. The interaction of the C-terminus with an adjacent PA domain aids crystal packing, the protomers are linked together, akin to a chain (Supplementary Figs. 6a,b). A similar observation was also seen in PaAP_T_E340A(trunc)_ (Supplementary Fig. 6c), which instead packed head to tail.

### Kinetic analysis of PaAP

Purified recombinant PaAP, which encompassed residues 27–536 relating to the FL-secreted protein (that is, lacking the N-terminal signal peptide), was initially used. To assay the activity of PaAP, we employed the well-established aminopeptidase assay with p-nitroanilide (pNA) coupled amino acids as substrates. Full-length PaAP had low activity (Fig. 1d and Supplementary Fig. 7a), consistent with previous reports that PaAP is secreted as a 58 kDa propeptide (Pro-PaAP), which requires C-terminal processing to yield an active enzyme^24^. As a result, we truncated the C-terminus (residues 513–531) by incorporating a C-terminal thrombin cleavage site. This allowed the selective and efficient removal of the C-terminus during purification to produce PaAP_T (a C-terminal truncated form of PaAP). The thrombin cleavage site avoided complications that can arise when attempting to overexpress and purify a constitutively active aminopeptidase from the cytoplasm of *Escherichia coli* cells. PaAP_T was highly active in comparison to full-length PaAP (an increase of ~100-fold in *k*_cat_, while *K*_M_ remained unchanged), suggesting the C-terminus is involved in suppressing PaAP activity, in agreement with previous studies^23,24^.

The kinetic activity of PaAP was thoroughly investigated by examining the effect of pH, the requirement for metal ions and the evaluation of catalytic mechanism using active site mutants (Fig. 1d and Supplementary Fig. 7). The substrate specificity was also probed by testing the activity of PaAP_T against several commercially available amino acid–pNA substrates (Supplementary Fig. 7b). All substrates had similar *K*_M_ values, whereas *k*_cat_ values varied considerably. The *k*_cat_ was highest for Leu-pNA (689 min^−1^), followed by Lys-pNA (248 min^−1^), while the other substrates tested had much lower *k*_cat_ values (for example, 6.9 min^−1^ for Val-pNA). The efficiency of the enzyme (*k*_cat_/*K*_M_) for each substrate suggests Leu-pNA and Lys-pNA as the most preferable substrates.

### PaAP cleaves peptides both processively and distributively

The amino acid–pNA assay is a convenient and simple direct assay; however, the small peptide mimic is less complex and possesses a p-nitro aniline leaving group, not comparable to an amino acid or peptide. Therefore, we developed a discontinuous assay using peptide substrates to better understand the activity and mechanism of PaAP. Several peptides, varying in length and composition, were incubated with PaAP overnight in a standard reaction buffer, alongside a control (PaAP omitted). The samples were analyzed by liquid chromatography–mass spectrometry (LC–MS) or matrix-assisted laser desorption/ionization (MALDI); samples were deemed to be substrates if there was a mass shift between the control and those incubated with PaAP (Supplementary Figs. 8 and 9). Our results demonstrated that PaAP was capable of processing peptides of varying lengths (2 amino acid up to 25 amino acid) and composition (Supplementary Figs. 9–12). This includes a casein-derived peptide (α-casein(90–95), RYLGYL) and its own C-terminus (ERWGHDFIK) and unstructured N-terminus (Supplementary Fig. 10). However, more complex peptides (containing secondary structure and disulfide bonds), such as human defensins, were not processed (Supplementary Fig. 11). Peptides with modified termini (N-terminal acetylation and C-terminal amidation) were tested as substrates. While a peptide with an acetylated N-terminus was not a substrate for PaAP (Supplementary Fig. 9h), a peptide harboring a C-terminal amide (ERWGHDFIK-NH_2_) was a substrate (Supplementary Fig. 13c).

**Fig. 2:**
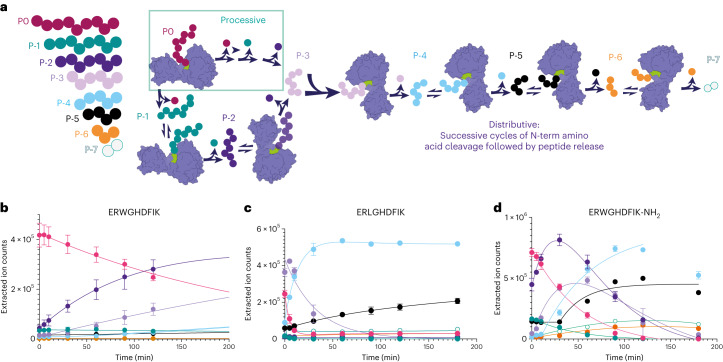
Kinetics of peptide degradation catalyzed by PaAP. **a**–**d**, Time courses are shown for different peptide substrates. **a**, Schematic of a fully distributive reaction where PaAP releases a product every reaction cycle (left) or a mixed reaction where the first amino acids from the N-terminus are removed in a processive manner so that for the first hydrolysis step(s), products remain bond to the enzyme and undergo next amino acid hydrolysis event. **b**, Time course for ERWGHDFIK degradation. **c**, Time course for ERLGHDFIK degradation. **d**, Time course for ERWGHDFIK-NH_2_ degradation. Lines are fitted to single (P0 peptide) or double exponential equations (to reflect the formation and decay of intermediates). All raw data are shown in Supplementary Fig. 13 for these peptides, other peptides are shown in Supplementary Fig. 12 and fits in Supplementary Table 5. Experiments were carried out in triplicate, and errors are shown as standard error of the mean. Source data

To investigate the activity of PaAP further, we performed a time-course assay with a selection of peptide substrates. The initial substrate was broken down sequentially into smaller peptide products which could be detected by LC–MS using extracted ion chromatograms for each peptide product/intermediate. The time course showed a complex pattern in which parent peptides decreased over time, while intermediates (akin to truncation products) appeared and were consumed over time. This suggests that after cleaving the first N-terminal amino acid (P0), PaAP could use the product (P-1) as a substrate for further rounds of hydrolysis, and the process occurred for subsequent peptide intermediates. A schematic depicting the breakdown of peptides is shown in Fig. 2a. Because the pattern and rates of formation and decay of each peptide intermediate are different for peptides with varying sequences, we concluded PaAP does not exclusively function processively. A processive enzyme can catalyze consecutive reaction cycles without releasing its substrate. In contrast, a distributive enzyme releases a product every reaction cycle^25^. Curves for ERWGHDFIK, ERLGHDFIK and ERWGHDFIK-NH_2_ are shown in Fig. 2b–d, respectively, while curves for KWLGYL, KA-AMC (Lys-Ala-methylcoumaryl-7-amide), HCATIPAFDG and RWGHDFIK are shown in Supplementary Fig. 12a–d, respectively.

We hypothesized that the PA domain may be involved in binding and presenting substrates to the active site during catalysis besides its regulatory role. We examined the degradation of a peptide with a C-terminal amide (ERWGHDFIK-NH_2_). Surprisingly, ERWGHDFIK-NH_2_ was processed at a faster rate than unmodified ERWGHDFIK. We also performed time courses with the PaAP_T R189A variant, which degraded both ERWGHDFIK and KWLGYL peptides slower than PaAP_T (Supplementary Figs. 12a,e). Moreover, the R189A variant accumulates mid-stage breakdown products that had been completely turned over by PaAP_T (WGHDFIK and GHDFIK) after 24 h (Supplementary Fig. 12e). This reduction in rate and accumulation of mid-stage breakdown products suggests R189 may have a role in binding and/or catalysis of peptide substrates.

Comparison of time courses with different peptide substrates (Fig. 2b–d and Supplementary Figs. 12a–d and 13a–c) reveals a distinct pattern of substrate consumption and accumulation of intermediates. Fitting data to exponential equations revealed that while some substrates behave exclusively in a distributive manner (RWGHDFIK), meaning each reaction cycle releases a peptide product which then competes with other potential substrates for enzyme binding and for the next catalytic cycle, others did not. In a processive reaction, peptide intermediates do not accumulate over time, as there is one binding event for P0 followed by peptide bond cleavage without product release in subsequent cycles. Here for most peptides investigated, the first few amino acids were removed likely in a processive manner, because intermediates for those were not observed during the time course. After a few amino acids were removed, intermediates accumulated. For example, P-1 did not accumulate for ERWGHDFIK and ERWGHDFIK-NH_2_, but all later products (P-2 to P-7) accumulated later on. Alternatively, ERLGHDFIK did not accumulate P-1 or P-2 but did accumulate products from P-3 to P-7. HCATIPAFDG mainly accumulated products from P-5. This is consistent with a mixed system, in which the first few amino acids are removed processively and subsequent amino acids removed in a distributive manner. Processive kinetics was observed in a Xaa-Pro aminopeptidase involved in protein degradation in yeast^26^.

### Proteolysis of proteins

Previous studies using refolded PaAP determined it could perform autoprocessing in *cis*, meaning one molecule of PaAP could cleave its own N-terminal residues, but not the N-terminal residues from another PaAP molecule^24^. We hypothesized that PaAP could prune residues from proteins with accessible N-termini because peptides of longer lengths were substrates. To test this, we incubated PaAP with several proteins with known ordered and disordered N-termini and analyzed them by intact protein mass spectrometry (Supplementary Fig. 14). PaAP preferentially removes the N-terminal methionine residue from a few of the proteins tested (WP_010598044 and WP_077070634). In one instance, PaAP was able to liberate a few residues (GMQQ) from the N-terminus of BtCDPS up to the start of the known secondary structure (PDB: 6ZTU). By comparing the intact mass of PaAP constructs, we determined that the N-terminus of PaAP is autoprocessed in the active protein (Supplementary Fig. 10). Although PaAP could cleave the unstructured N-termini of some proteins, its own N-terminal processing likely occurred in *cis* as previously described^24^.

### Structure-guided inhibitor design

**Fig. 3:**
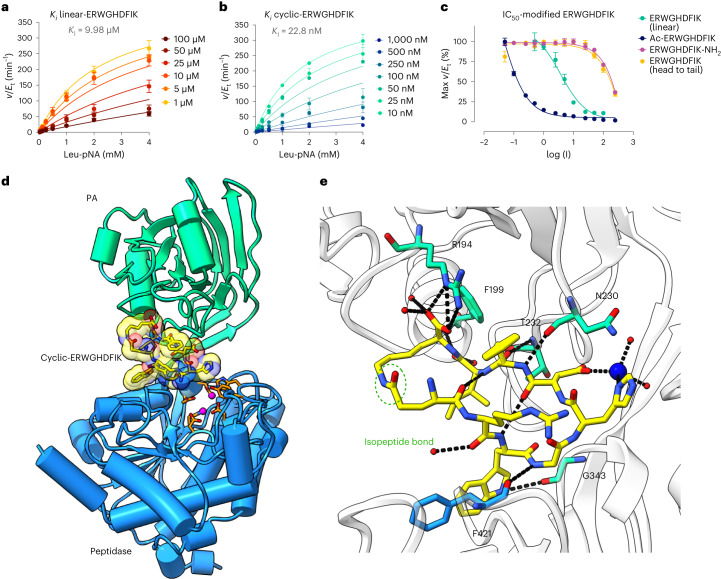
Structure-guided inhibitor design. **a**, Michaelis–Menten plot of PaAP_T in presence of increasing linear-ERWGHDFIK concentration. **b**, Michaelis–Menten plot of PaAP_T in presence of increasing cyclic-ERWGHDFIK concentration. The *K*_I_ of cyclic-ERWGHDFIK is ~440-fold lower than the *K*_I_ of linear-ERWGHDFIK. **c**, IC_50_ plot for linear-ERWGHDFIK (green) in comparison to N-terminally (acetylated, blue) and C-terminally (amidated, magenta) modified peptides, as well as peptide cyclized ‘head-to-tail’ (dark yellow). Datapoints show the mean of three independent measurements (*n* = 3) and s.d. **d**, Structure of PaAP_T bound to cyclic-ERWGHDFIK inhibitor. Cyclic-ERWGHDFIK (yellow) binds between the PA domain (turquoise) and the peptidase domain (blue) directly above the active site (orange), similar to the C-terminus in WT PaAP structure. **e**, Hydrogen bond network involved in binding cyclic-ERWGHDFIK. The side chain of R194 makes an important interaction with the carboxyl group. Source data

The activity of PaAP is proposed to complement other secreted proteases, *P. aeruginosa*, and recycle nutrients. Additionally, PaAP is abundantly expressed by isolates from cystic fibrosis lungs and promotes vesicle association^27^. Therefore, we used our structural findings to develop an inhibitor that mimicked the self-inhibitory conformation adopted by the full-length protein. We first tested a linear peptide (linear-ERWGHDFIK), based on the C-terminus of PaAP. We hypothesized that linear-ERWGHDFIK would inhibit PaAP_T activity (active form). Indeed, linear-ERWGHDFIK inhibited the activity of PaAP in the Leu-pNA assay with a *K*_I_ of 9.98 µM (Fig. 3a). However, linear-ERWGHDFIK would not be a viable inhibitor as we previously showed it also acts as a substrate for PaAP. Guided by the structure of PaAP, we designed a cyclic peptide (cyclic-ERWGHDFIK) cyclized via an isopeptide bond between the glutamate and lysine side chains. Cyclization between the side chains was preferred to preserve the carboxy terminus of the lysine residue and introduce minimal stress on the backbone angles. Cyclic-ERWGHDFIK was a potent inhibitor of PaAP with a *K*_I_ of 22.8 nM, which is ~440-fold lower than the linear peptide (Fig. 3b). Moreover, cyclic-ERWGHDFIK was not degraded when incubated overnight with PaAP (Supplementary Fig. 15). Other variations of the C-terminal peptide were tested as inhibitors (Fig. 3c). Those lacking the carboxyl group at C-terminus (that is, ERWGHDFIK-NH_2_ and cyclized head to tail) were very poor inhibitors.

The structure of PaAP_T in complex with cyclic-ERWGHDFIK (PaAP_T_(ERWGHDFIK)_) was solved to confirm enzyme–inhibitor interactions. The structure of PaAP_T_(ERWGHDFIK)_ is comparable to the structure of full-length PaAP (Fig. 3d), adopting the same closed/inactive conformation (RMSD = 0.3610 Å over 934 residues). Cyclic-ERWGHDFIK was predictably bound in the groove between the PA and peptidase domain, almost identical to the C-terminus bound in the full-length structure (Fig. 3e). Complete electron density was visible for the inhibitor, including the side chain linkage between K and E, which is shown in the omit map in Supplementary Fig. 6c. The inhibitor complex structure suggests inhibitor binding induces a conformational change, stabilizing the closed conformation and blocking access to the active site. Inhibition showed no time dependence (Supplementary Fig. 16), pointing toward this conformational change being a single-step process.

Next, we hypothesized the interaction between R194 and the peptide carboxy terminus to be important for inhibition. To test this, we produced the PaAP variant R194A to eliminate a likely salt bridge between the peptide COOH and the guanidinium of the protein arginine side chain. Indeed, the IC_50_ for linear-ERWGHDFIK with R194A variant was determined as 306 μM, a 100-fold increase compared to full-length PaAP. Furthermore, no inhibition was detected for cyclic-ERWGHDFIK within the inhibitor range tested (0.05–100 μM), and an IC_50_ value could not be determined. This suggests that the interaction between R194 and the carboxy terminus is crucial for inhibition, in contrast to the little effect seen on substrate binding. In addition, the ERWEGHDFIK-NH_3_ peptide was not an inhibitor of PaAP_T within the concentration of peptide tested, although it was shown to be a good substrate. This finding suggests that the carboxy terminus is a key for the interaction with the PA domain, which likely has a regulatory function.

### PaAP expression and activity impact growth and biofilms

PaAP was previously reported to have a role in biofilm development and growth on a complex nutrient source. A variant carrying a PaAP deletion (Δ*PaAP*) showed increased propidium iodide staining, moderately reduced CFU per ml in a pellicle biofilm assay and reduced growth in minimal medium with casein as a sole carbon and nitrogen source^28^. We generated a clean deletion of the *PA14_26020* locus in the UCBPP-PA14 strain background of *P. aeruginosa (*Δ*PaAP*) to determine the baseline behavior in the complete absence of PaAP activity, and also reintroduced the *pa14_26020* gene under the control of the arabinose-inducible P_*BAD*_ promoter at the *attB* locus(P_*BAD*_-*PaAP)*. We then investigated the effects of modulating PaAP expression levels or inhibiting its activity using the cyclic-ERWGHDFIK peptide on cellular phenotypes in *P. aeruginosa*.

**Fig. 4:**
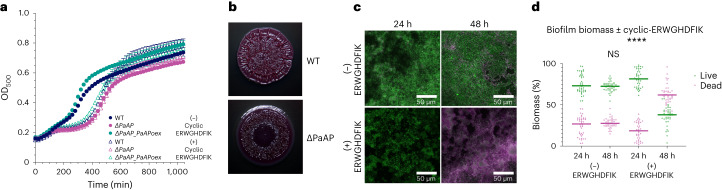
PaAP in vivo assays. **a**, Growth curve of WT *P. aeruginosa* UCBPP-PA14 (blue) in comparison to *ΔPaAP* (PaAP deletion variant, pink) and *ΔPaAP_PaAPoex* (PaAP under the control of an arabinose-inducible PBAD promoter, turquoise). Absorbance at OD_500_ was used to monitor growth in casein medium supplemented with arabinose. Growth was carried out in the presence (no fill circle) and absence (solid fill circle) of cyclic-ERWGHDFIK inhibitor. *ΔPaAP* variant has a growth defect compared to WT, while *ΔPaAP_PaAPoex* has a growth advantage compared to WT in the absence of inhibitor. The inhibitor suppresses growth of WT and *ΔPaAP_PaAPoex* to the level of Δ*PaAP*. Datapoints show the mean of four independent measurements (*n* = 4) and standard error. **b**, PaAP deletion shows a biofilm development phenotype when grown on a solid medium. Representative images are shown of *n*_biological_ = 4 biological replicates. **c**, Representative images of Live/Dead BacLight-stained 24- and 48-h-old biofilms, grown with (+) and without (−) cyclic-ERWGHDFIK. Merge of green (live cells) and magenta (dead cells) channels shown. **d**, The percentage of total biomass calculated for both channels (green, live cells; magenta, dead cells) is plotted. In the presence of cyclic-ERWGHDFIK, there are significantly more propidium iodide-stained (dead) cells after 48 h compared to untreated. Supplementary Fig. 17 shows raw data for all growth curves. Significant differences were assessed through an unpaired Mann–Whitney test (two-sided). *P* = 0.0548, ****P < 0.00001. *n*_biological_ = 4 biological replicates with ten random z-stack images (40 datapoints). NS, not significant. Source data

First, we examined the impact of PaAP expression and activity on growth when casein was the sole source of carbon and nitrogen (Fig. 4a). As expected, the *ΔPaAP* strain showed a substantial growth defect in this medium. The P_*BAD*_**-***PaAP* strain grew slightly better than the *ΔPaAP* strain even in the absence of arabinose, likely due to leaky expression from the P_*BAD*_ promoter. The addition of arabinose caused growth of the P_*BAD*_**-***PaAP* strain to surpass that of the wild type (WT), suggesting that PaAP expression and activity may be limiting for growth under this condition. In the presence of 10 µM cyclic-ERWGHDFIK and arabinose, growth of both the WT and the P_*BAD*_**-***PaAP* strain were inhibited (although not to the same degree as the *ΔPaAP* strain), while the *ΔPaAP* strain was unaffected (Supplementary Fig. 17). In the presence of 100 µM cyclic-ERWGHDFIK, the WT and P_*BAD*_-*PaAP* strain grew only slightly more than the *ΔPaAP* strain, indicating nearly complete inhibition of PaAP for providing amino acids from casein to support growth (Supplementary Fig. 17).

Next, we investigated the roles of PaAP in a more complex biofilm context. We grew the WT and *ΔPaAP* strains on 1% tryptone agar supplemented with Congo Red and Coomassie Blue to observe colony morphology, as described previously^29^. We observed subtle differences, with reduced wrinkling of the *ΔPaAP* strain in the center of the colony, supporting a role for PaAP activity in organization and development of biofilm structure (Fig. 4b). Finally, we repeated the propidium iodide staining of pellicle biofilms that previously showed increased staining for a *ΔPaAP* strain in the PAO1 background^28^. As expected, we also observed increased propidium iodide staining in our *ΔPaAP* strain in 48-h old biofilms (Supplementary Fig. 18). Notably, WT biofilms grown in the presence of cyclic-ERWGHDFIK also increased propidium iodide staining after 48 h to a similar extent as the *ΔPaAP* strain, consistent with substantial inhibition of PaAP activity even in this complex environment and over a long time period (Fig. 4c,d). Greater than 60% peptide remained in solution after 48 h growth, demonstrating the available compound after an extended period of biofilm growth (Supplementary Fig. 19).

### PaAP is largely under purifying (negative) selection

It is possible to estimate the strength of selection a protein is under by comparing synonymous substitution rates (dS)—assumed to be neutral—with nonsynonymous substitution rates (dN). Nonsynonymous substitution rates are exposed to selection as they change the amino acid composition of a protein. Using the FUBAR analysis package^30^, we analyzed nucleotide sequences for *paaP* from all 2,887 *P. aeruginosa* genome sequences available in the NCBI genome database with an assembly level of at least ‘scaffold’. For comparison, we performed a similar analysis on the *lasB* and *lasR* nucleotide sequences from the same set of genomes. lasR is frequently mutated among clinical isolates^31^, while on the other hand, *lasB* has been described as important for fitness in infection contexts^13^. The dN/dS analysis revealed that PaAP had 108 amino acids with evidence of negative selection for change, and one amino acid with positive selection, indicating overall PaAP tolerates mutations poorly. Similarly, for LasB, there were 121 sites under negative selection and 2 sites under positive selection. For LasR there were 16 sites under negative selection and 17 sites under positive selection. Supplementary Fig. 20 shows amino acid positions under negative or psubstrates agree somewhat with previous ositive selection.

## Discussion

For bacteria living in dense, complex communities, secreted proteases are a double-edged sword. Their activities can help efficiently recycle or access complex nutrients^19^, neutralize proteinaceous threats^32^ and control the architecture of the extracellular matrix in which cells are often embedded^33^. However, their production can be costly to individual cells, and their activity can be dangerous if not tightly controlled. Here, using structural and biochemical approaches, we interrogated the molecular mechanisms of activity and regulation for the secreted aminopeptidase, PaAP. Our work has revealed new insights into the range of substrates on which it can act and its post-translational regulation, leading to the rational design of a potent inhibitor.

Until this study, all investigations into the activity of PaAP were conducted with small molecule substrates, consisting of amino acids conjugated to a chromophore or fluorophore^23,34^. Although these substrates provide a convenient way to assay peptidase activity, they employ minimalistic substrate mimics and do not necessarily recapitulate trends observed with peptide substrates. Our results using amino acid–pNA substrates agree somewhat with previous reports, where Leu and Lys-pNA are preferred over other amino acids^23^. However, the protein used in previous studies was not homogeneous and multiple species were present depending on extracellular processing^18,23,24,35^. Instead, we purified recombinant PaAP and variant forms, ensuring enzyme homogeneity. Moreover, we tested the activity of PaAP against a variety of peptide substrates. We observed the removal of amino acids from the N-terminus of peptides over time by LC–MS. Some peptide intermediates accumulated to a larger extent than others (those with Trp or His at the N-terminus) suggesting that these residues are unfavorable substrates. Ultimately, these peptide intermediates with N-terminal Trp or His residues were also degraded. This was also exemplified by the ERLGHDFIK peptide (W replaced by L), which was degraded at a faster rate than ERWGHDFIK. We tested PaAP activity against different protein substrates and concluded PaAP can liberate amino acids from proteins with accessible N-termini, although not all proteins tested could be processed (Supplementary Fig. 14). Moreover, it appears that PaAP preferentially removes the N-terminal methionine from these proteins. However, in two cases, additional residues were removed. These include the removal of GMQQ from the N-terminus of BtCDPS and the autoprocessing of PaAP’s own N-terminus to remove GSEAQQFTEFW. Autoprocessing was identified by comparing intact mass spectrometry results between active PaAP_T and inactive PaAP_T_E340A_, whereby a ten-amino-acid sequence is cleaved in PaAP_T. Interestingly, the thrombin-cleaved C-terminus of PaAP_T_E340A_ (confirmed by intact MS) remained as a bound ligand in the crystal structure, suggesting that the liberated C-terminus propeptide is degraded by active PaAP.

Although PaAP shows substantially increased activity after C-terminal tail removal, full-length protein is catalytic active, with lower *k*_cat_. The substrate Leu-pNA and the competitive inhibitor cERWGHDFIK bind to the same enzyme form, as it must be for competitive inhibition to occur. Productive substrate binding to the closed conformation is unlikely given the active site and metal occlusion when C-terminal tail occupies the active site and inhibits PaAP. It is likely, therefore, that the full-length protein is sampling open (active, C-terminal tail away from the active site) and closed (inactive, C-terminal tail occupying the active site) conformations when free in solution.

PaAP transcription is tightly regulated. It is directly affected by three global regulators, which are as follows: the stress sigma factor RpoS^22^, the QS regulator LasR^21,36^ and an RNA polymerase-binding protein that is upregulated under anoxic conditions, SutA^37^. All of these regulators are active in the high-density, nutrient- and oxygen-limited context of a biofilm, where total levels of gene expression activity may be low but efficient utilization of limited resources is important. As a result, PaAP is one of the most abundant proteins in the biofilm matrix and in OMV, which are a major component of the *P. aeruginosa* biofilm matrix^17,27^. PaAP has been proposed to function in biofilm development^28^, biofilm remodeling^7^ and acquisition of nutrients from complex sources^19^. As a result, PaAP has been termed a ‘public good’ enzyme, secreted by some members of the biofilm to benefit the bacterial community more broadly^19^. Because *P. aeruginosa* forms high-density aggregates in many environments, including infections, a better understanding of factors contributing to metabolism and developmental decisions in such contexts is needed.

**Fig. 5:**
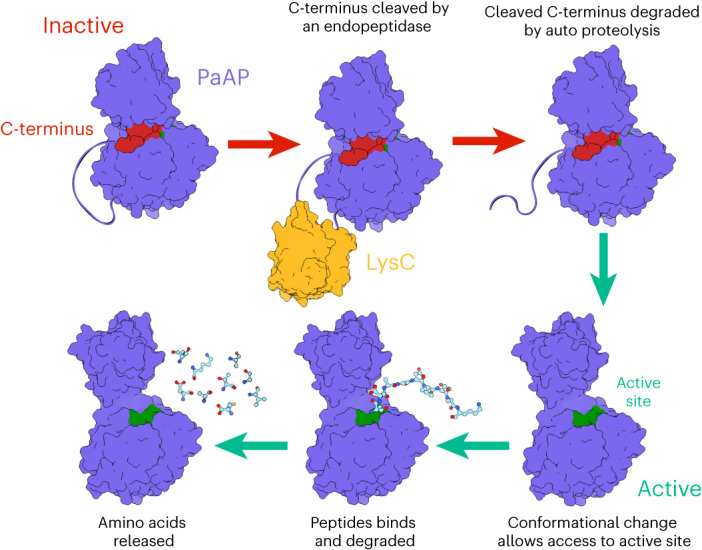
Summary of PaAP activation. PaAP is secreted from the cell as an inactive enzyme. The C-terminus is a propeptide sequence that binds between the PA and peptidase domains, inducing a closed inactive conformation where the active site is occluded. A disordered, protease-accessible region linking the C-terminus to the peptidase domain is nicked, which allows the propeptide to be released and degraded. Removal of the C-terminus propeptide induces a conformational change where the PA domain rotates outwards, exposing the active site. Activated PaAP can then degrade unstructured peptides, releasing amino acids from the N-terminus.

PaAP activity is also post-translationally regulated (Fig. 5). It is first secreted by the type II secretion system, which is also under QS control^4,38^. A further level of regulation is imposed by the C-terminal propeptide sequence of PaAP, which must be proteolytically removed^24^. Removal of the C-terminus is believed to be performed by LysC, although elastase and alkaline phosphatase may also be involved, by indirect activation of LysC^23^. Here we demonstrate, at the molecular level, how the C-terminal propeptide regulates the activity of PaAP. The structure of PaAP reveals the C-terminus binds in a groove between the PA and peptidase domains, which blocks access to the active site and locks these two domains into a closed conformation. When the C-terminus was truncated, PaAP adopted an open conformation, where the PA domain is free to rotate, uncovering the active site. We also considered that the PA domain may be involved in binding and delivering peptides into the active site, analogous to PDZ domain function in carboxy-terminal processing proteases^32^, due to its propensity to interact with the carboxy terminus of peptides in our different crystal forms. Indeed, the PaAP_T_R189A_ variant showed a modest reduction in the rate of peptide degradation but retained a similar *k*_cat_ upon the minimal Leu-pNA substrate. Additionally, ERWGHDFIK degradation accumulates the intermediates WGHDFIK and GHDFIK, when incubated with PaAP_T_R194A_, but not the WT enzyme. In contrast, the ERWGHDFIK C-terminus NH_2_-modified peptide degraded faster than the peptide containing the free carboxyl group. This could be due to ERWGHDFIK-NH_2_ peptide not interacting with PaAP as an inhibitor, while linear-ERWGHDFIK does. This is supported by the increased IC_50_ value of ERWGHDFIK-NH_2_ compared to ERWGHDFIK. Moreover, N-terminally modified Ac-ERWGHDFIK (which is not a substrate) is a stronger inhibitor (lower IC_50_) than an unmodified linear substrate. This regulatory mechanism involving the C-terminus propeptide and the PA domain is likely to be a common feature of this class of aminopeptidases. The structure of a homolog deposited to the PDB (6HC6) shows a similar interaction between the C-terminus and the PA domain to what is seen in PaAP, suggesting that the insight gained here is generalizable to secreted aminopeptidases from other organisms (Supplementary Fig. 1).

The phylogenetic analysis shows that PaAP and LasB are highly conserved and do not seem to tolerate a large number of mutations; it additionally indicates that in existing natural contexts there is pressure to maintain PaAP sequence, and therefore self-inhibition and activation, unchanged.

We rationally developed a potent inhibitor of PaAP based on the interaction between the C-terminus propeptide (ERWGHDFIK) and the PA and peptidase domains. The peptide was cyclized to increase stability and decrease conformational flexibility^33^. Indeed, cyclic-ERWGHDFIK was not cleaved by PaAP after prolonged incubation (Supplementary Fig. 15). In addition, cyclic-ERWGHDFIK was far more potent with a *K*_I_ of 22.8 nM. Although higher concentrations were required in the more complex environments of bacterial growth and biofilm assays (where cell density is high), we were able to show that the inhibitor affected phenotypes in liquid media and biofilms similar to the deletion variant lacking PaAP (Fig. 4).

PaAP has recently attracted attention as a complementary enzyme to other well-characterized major virulence factors. PaAP is one of the most abundant OMV proteins and is implicated in association of vesicles with lung cells^7,27^. OMVs have diverse roles but are believed to contribute to deployment of virulence factors and nutrient acquisition for *P. aeruginosa* in infections^8^. Given its demonstrated role in facilitating growth on a protein substrate (Fig. 4a), PaAP may be a dominant contributor to the nutrient acquisition function of OMVs.

Antivirulence therapy is a relatively new strategy that has the ability to control the emergence and spread of resistant pathogens^39–41^. Several aminopeptidases have been described as virulence factors in multiple organisms. Examples are the arginine-specific aminopeptidase from *Pseudomonas*^42^, PepP from *Campylobacter jejuni*^43^ and aminopeptidase T from *Listeria monocytogenes*^44^. Moreover, the leucine aminopeptidase from *Staphylococcus aureus* has been investigated as a drug target, where its suppression led to improved mouse survivability^45^. To our knowledge, only the commercially available drug, Balsalazide, has impacted the activity of PaAP in vivo, where it displayed an anti-biofilm phenotype^34^. Here we demonstrated that our inhibitor, cyclic-ERWGHDFIK, also impacted pellicle biofilm development and growth on complex media. This demonstrates the target engagement of PaAP in a biofilm context, given that the ΔPaAP biofilm phenotype was phenocopied by peptide administration.

In conclusion, we have extensively characterized the extracellular PaAP. We have shown the molecular mechanism by which the C-terminal propeptide domain regulates the activity of PaAP. We developed a potent inhibitor of enzyme activity in vitro, which also impacted *P. aeruginosa* growth in late-stage biofilms and in nutrient-specific media similar to a *ΔPaAP* strain.

## Methods

### Materials, chemicals and peptide analytical data

The chemicals used in this investigation were purchased from Fisher Scientific and Sigma-Aldrich. Peptides tested as substrates and inhibitors (ERWGHDFIK, ERWGHDFIK cyclized head-to-tail, cyclic-ERWGHDFIK cyclized by isopeptide bond, Ac-ERWGHDFIK, ERWGHDFIK-NH_2_ and ERLGHDFIK) were commercially obtained from Peptide Synthetics. Data supporting the purity of synthesized peptides are provided in the supplementary information section (Supplementary Note). Peptides were obtained at higher than 90% purity by HPLC and the identity was confirmed by mass spectrometry. Analysis was carried out using a 100 Å 4.6 × 50 mm column, gradient from 0–80% acetonitrile, in 8 min at 60 ºC. Flow rate: 1.5 ml min^−1^, injection volume: 20 µl; column and Kinetex XB-C18 2.6 µ 100 A. The peptide was purified in acetonitrile and water containing 0.1% trifluoroacetic acid before lyophilization.

Longer peptide substrates (HCATIPAFDG, VTATIAFPAYDGE and VGAGIGWPWTAEHVDQTLASGNDIC) were a kind gift from James Naismith (Oxford University). Peptides RYLGYL (α-casein(90–95)), β-defensin 1 Human and β-defensin 2 Human were purchased from Sigma. Primers and DNA fragments used for cloning were from IDT, and primer sequences are available in Supplementary Table 3. Kinetic data were fitted using GraphPad Prism.

### Strains, media and growth conditions

Supplementary Table 4 shows a summary of all strains used. The clean deletion of *PA14_26020* from the UCBPP-PA14 genome was carried out as previously described with minor variations^46^. Briefly, 600 bp upstream and downstream of the gene were cloned into the pMQ30 suicide vector using Gibson assembly, and this was introduced by conjugation from *E. coli* into *P. aeruginosa*, where it is integrated by homologous recombination into the genome adjacent to *PA14_26020*. Subsequently, colonies that lost the plasmid and the WT copy of the gene by homologous recombination were selected by plating on sucrose and confirmed by colony PCR using check primers that flanked the gene. To reintroduce the *PA14_26020* gene sequence under the control of the arabinose-inducible P_*BAD*_ promoter at the *attB* site, the coding sequence and ribosome binding site for *PA14_26020* were cloned into a previously described pUC18-miniTn7T-Gm^R^ variant encoding the P_*BAD*_ promoter using Gibson assembly. This plasmid was then conjugated from *E. coli* into *P. aeruginosa* along with a helper strain to catalyze recombination into the *attB* site as described previously^47^ (see Supplementary Table 3 for primers).

### Media and growth conditions

Lysogeny broth (LB) contained 10 g l^−1^ NaCl, 10 g l^−1^ tryptone and 5 g l^−1^ yeast extract, solidified with 15 g l^−1^ agar for solid media. LB was supplemented with 100 µg ml^−1^ carbenicillin, 20 µg ml^−1^ gentamicin or 50 µg ml^−1^ kanamycin as appropriate for the selection of plasmids in *E. coli*. Phosphate-buffered minimal media with casein as a sole carbon and nitrogen source contained 35.9 mM K2HPO4, 14.2 mM KH2PO4, 42.8 mM NaCl, 1.0 mM MgSO4, 0.1 mM CaCl_2,_ 0.5% (wt/vol) casein from bovine milk (Sigma-Aldrich, C5890) and trace metals (7.5 μM FeCl_2_·4H_2_O, 0.8 μM CoCl_2_·6H_2_O, 0.5 μM MnCl_2_·4H_2_O, 0.5 μM ZnCl_2_, 0.2 μM Na_2_MoO_4_·2H_2_O, 0.1 μM NiCl_2_·6H_2_O, 0.1 μM H_3_BO_3_ and 0.01 μM CuCl_2_·2H_2_O). Media was sterile-filtered with a 0.2 μm filter but also autoclaved for 15 min to achieve solubilization of the casein. This may have resulted in some partial degradation of casein. Cultures were incubated at 37 °C with shaking unless otherwise indicated.

### Cloning, mutagenesis and constructs

The gene encoding PaAP (*PA2939* (PAO1 genome)/*PA14_26020* (UCBPP-PA14 genome)) from *P. aeruginosa* was synthesized as a gBlock from IDT and cloned into the pJ414 vector in-frame with a TEV cleavable N-terminal His_6_ tag using the Gibson assembly method. PaAP was cloned without the N-terminal signal peptide sequence (MSNKNNLRYALGALALSVSAASLAAP), so the full-length PaAP construct in this study starts from Serine 27. Mutant and truncated proteins were produced by standard site-directed mutagenesis. Constructs and mutations were confirmed by sequencing before transformation into the *E. coli* expression strain SHuffle T7 by NEB. The following five constructs of PaAP were used in this study: PaAP (WT, residues 27–536); PaAP_T (Thrombin cleavage site (LVPRGS) substituted into position 513–518, thrombin used to remove C-terminus); PaAP_T_E340A_ (E340A variant incorporated into PaAP_T construct); PaAP_T_E340A(trunc)_ (stop codon TGA incorporated into PaAP_T_E340A_ construct, so the C-terminus was truncated without requiring thrombin cleavage). A list of primers used to create constructs can be found in Supplementary Table 3.

### Protein production and purification

Cells transformed with PaAP constructs were grown at 37 °C (shaken at 180 rpm) in LB media supplemented with 100 μg ml^−1^ ampicillin until an OD_600_ of 0.8 was reached. Gene expression was induced with 1 mM isopropyl β-d-1-thiogalactopyranoside and incubated overnight at 18 °C (shaken at 180 rpm). Cells were collected by centrifugation at 8,855*g* (Beckman JLA 8.1000 rotor) for 10 min and the pellets were stored at −20 °C. Cells were resuspended in fresh lysis buffer (50 mM HEPES, pH 8.0; 20 mM Imidazole, pH 8.0, 250 mM NaCl) and incubated with ~1 mg ml^−1^ lysozyme for 30 min at 4 °C. Cells were lysed using a high-pressured cell disruptor. Insoluble cell debris was removed by centrifugation for 30 min at 33,000*g* (Beckman JA 25.50). The supernatant was loaded onto a 5 ml HisTrap HP column (GE Healthcare) pre-equilibrated with lysis buffer. The column was washed with 20 column volumes (CV) of lysis buffer and 10 CV 10% elution buffer solution (50 mM HEPES pH 8.0, 250 mM NaCl, 300 mM Imidazole pH 8.0) to remove nonspecific interacting proteins. Ten column volumes of elution buffer was pasted over the column to elute the His_6_-tagged PaAP. Fractions containing PaAP were pooled and an appropriate amount of TEV protease was added (1:50, PaAP:TEV) to remove the N-terminal tag. In constructs containing an incorporated thrombin cleavage site, thrombin was added (1:100, PaAP:thrombin), to remove the C-terminus. Pooled protease-treated samples were dialyzed overnight against 2 l of dialysis buffer (25 mM Tris, pH 8.5, 200 mM NaCl). Cleaved protein was separated from residual tagged protein by a second passage over the HisTrap column. Flow through and wash solution containing unbound protein were concentrated to 5 ml and injected onto a 10/16 S200 size exclusion chromatography column for polishing (and to remove thrombin contamination when used). Fractions containing PaAP were pooled and concentrated to 10 mg ml^−1^ for further experiments.

### Structure determination

Crystals were grown at 20 °C using the sitting drop vapor diffusion technique, with a drop size of 0.3 μl in a ratio of 2:1, reservoir:protein solution. A list of crystallization conditions is provided in Supplementary Table 2. Crystals were cryoprotected in mother liquor supplemented with 20% (v/v) ethylene glycol before being flash cooled in liquid nitrogen. Diffraction data were collected at the Diamond Light Source in Oxford, UK, on I03 and I04 beamlines. Data reduction and processing were completed using XDS and the xia2 suite. The structure was solved by molecular replacement with PHASER searching for two individual components (peptidase and PA domain). Search components comprised an ensemble of three PDBs (6HC6, 5IB9 and 1TKJ), which had been modified sculptor (using the CCP4 suite version 8.0) and manually truncated in COOT (version 0.8.9.2). Variant and ligand complex structures (PaAP_T, PaAP_T(ERWGHDFIK), PaAP_TE340A and PaaP_TE340A(trunc)) were solved using the structure of WT PaAP as the search model in PHASER. Protein structures were built/modified using COOT, with cycles of refinement in PHENIX. Metal ion selection and placement were made based on electron density, coordination, and X-ray fluorescence scan data (Supplementary Fig. 21), and were limited to elements found in crystallization conditions. Metal ion placement was verified by refinement and using the online server CheckMyMetal^48,49^. Crystallographic data are shown in Supplementary Table 1.

### Enzymatic assay with amino acid–pNA substrates

Aminopeptidase activity was determined spectrophotometrically primarily using amino Leu-pNA. Other commercially available pNA substrates (isoleucine, methionine, alanine, valine, proline, arginine, lysine and phenylalanine) were also assayed. Leu-pNA was incubated with enzyme (100 nM) in assay buffer (50 mM Tris, pH 8.5, 200 mM NaCl) at 25 °C. A pNA release caused an absorbance increase at 405 nm, monitored using a BMG Labtech plate reader. Kinetic constants *K*_M_ and *k*_cat_ were determined from assays varying substrate concentration (between 5 mM and 31.25 μM). Data were plotted in GraphPad Prism (version 9) and the Michalis Menten equation was used for fitting.

### Inhibitor studies

Inhibition assays were carried out with the following peptides: linear-ERWGHDFIK (PaAP C-terminal residues), cyclic-ERWGHDFIK, N-acetylated and C-amidated ERWGHDFIK. IC_50_ values were determined by varying peptide concentration at a fixed substrate concentration (fixed at *K*_M_, (S) = 2 mM, PaAP concentration was 100 nM). *K*_I_ was determined by assaying multiple fixed inhibitor concentrations (1,000 nM, 500 nM, 250 nM, 100 nM, 50 nM, 25 nM and 10 nM) at varying substrate concentrations under standard reaction conditions with Leu-pNA as a substrate (outlined above). *K*_I_ values were determined by fitting the data to equation (1) (Morrison’s equation):

1 $$v=1-\,\frac{\left({E}_{{\mathrm{t}}}+I+\,{K}_{{\mathrm{I}}}-\,\sqrt{{\left({E}_{{\mathrm{t}}}+I+{K}_{{\mathrm{I}}}\right)}^{2}-4{E}_{{\mathrm{t}}}I{K}_{{\mathrm{I}}}}\right)}{2{E}_{{\mathrm{t}}}}$$

where *v* is the velocity, *E*_t_ is the total enzyme concentration, *I* is the concentration of inhibitor and *K*_I_ is the equilibrium dissociation constant for the inhibitor. IC_50_ values increased when measured at increasing S, consistent with competitive inhibition.

### Peptide digestion assay

A variety of peptides of varying lengths and compositions were incubated with PaAP at room temperature. Assays were performed by incubating (overnight at room temperature) 500 μM of substrate (peptides) with 1 μM enzyme (PaAP) in reaction buffer (50 mM Tris, pH 8.5, 100 mM NaCl). The reactions were quenched by heating to 100 °C for 10 min followed by centrifugation at 17,000*g* in a benchtop centrifuge to remove precipitate. Supernatant was collected and samples were analyzed by LC–MS. Initial trial assays were carried out in the presence and absence (control) of enzyme to determine if the substrate was degraded (single or triplicate experiments). Time-course assays were performed on selected peptide substrates in triplicate. A 300 μl reaction (including 500 μM of substrate) was initiated by the addition of 1 μM enzyme. The reaction was incubated at room temperature. The reaction was quenched at set timepoints by the removal of 30 μl aliquots, which were immediately heated to 100 °C for 10 min. The samples were analyzed by LC–MS using a Xevo Q-ToF (Waters) as described below, searching for masses of all peptide breakdown products.

### Data analysis for enzyme kinetics

Peptide degradation time courses were fitted to exponential equations as follows:

P0 peptide consumption was fitted to a single exponential equation (equation (2)) with the format

2 $$y\left(t\right)=\,\left({y}_{0}-{{\mathrm{Plateau}}}\right){e}^{-{kt}}+{{\mathrm{Plateau}}}$$

Time courses for the formation and decay of peptide intermediates were fitted to double exponential equations (equation (3)) and single exponential equations, followed by a linear phase (4)

3 $$y\left(t\right)=\,\left({y}_{0}-{{\mathrm{Plateau}}}1\right){e}^{-k1t}+\left({y}_{0}-{{\mathrm{Plateau}}}2\right){e}^{-k2t}+\,{{\mathrm{Plateau}}}$$

4 $$y\left(t\right)=\,\left({y}_{0}-{{\mathrm{Plateau}}}\right){e}^{-{kt}}+{vt}+\,{{\mathrm{Plateau}}}$$

where *y*(*t*) is the product formed at time *t*, *y*_0_ − Plateau is the amplitude of the exponential phase, *k* is the observed rate constant for the exponential phase, *y*_0_ is the value of *y* at time 0, Plateau is the value of *y* after exponential phase, *C* is the offset and *v* is the slope of the linear phase.

Supplementary Table 5 details fitted parameters for each peptide species.

For pH dependence of kinetic parameters, data were fitted to two models, *n* = 1 accounting for a single ionizable group that needs to be deprotonated for binding/catalysis and *n* = 2 for two ionizable groups that need to be deprotonated for binding/catalysis. In equation (5), *y* is the kinetic parameter, *C* is the pH-independent value of *y*, pH is the experimental pH and pKa is the apparent acid dissociation constants for ionizing groups.

5 $$y={\rm{log}}\left(\frac{C}{\frac{1+\,{({10}^{-{{\mathrm{pH}}}})}^{n}}{{({10}^{-{{\mathrm{pka}}}})}^{n}}}\right)$$

### Protein digestion assay

A variety of proteins (1 mg ml^−1^), purified in-house, were incubated in the presence or the absence of PaAP (0.01 mg ml^−1^) overnight at room temperature in standard assay buffer (50 mM Tris, pH 8.5, 100 mM NaCl). After incubation, samples were analyzed by intact mass. Mass differences between ± PaAP samples were evaluated by sequentially truncating the N-terminus.

### Intact mass spectrometry analysis ESI

Protein samples were analyzed by intact mass spectrometry at the University of St. Andrews mass spectrometry and proteomics facility. The protein sample (10 µl and 1 µM) was desalted on-line through a MassPrep On-line Desalting Cartridge 2.1 × 10 mm, using a Waters Acquity H class UPLC plus, eluting at 200 µl min^−1^, with an increasing acetonitrile concentration (2% acetonitrile, 98% aqueous 1% formic acid to 98% acetonitrile and 2% aqueous 1% formic acid) and delivered to a Waters Xevo electrospray ionization mass spectrometer, which was calibrated using horse heart myoglobin. An envelope of multiply charged signals was obtained and deconvoluted using MaxEnt1 software to give the molecular mass of the protein.

### MALDI

Peptide samples incubated in the presence and absence of PaAP were initially analyzed by MALDI. The peptide sample (0.5 µl at 10 µM) was spotted along with matrix (0.5 µl 10 mg ml^−1^ α-cyano-4-hydroxycinnamic acid in ACN/0.1% TFA (50/50, vol/vol)) on a stainless steel MALDI plate and left to dry. The sample plate was loaded into a Sciex 4800 MALDI mass spectrometer. The laser voltage was adjusted to give a good signal to noise strength, and 50 shots were acquired in a random pattern across the spot and spectra combined. The spectra were externally calibrated with Sciex Pepcal mix (range: 500–4,000 m/z).

### Biofilm assay

Biofilms, formed at the air–liquid interface (pellicles), of WT *P. aeruginosa* PA14 were grown in an eight-well chambered coverglass slide (Nunc Lab-Tek II Chambered Coverglass). *P. aeruginosa* overnight culture was diluted 1/100 into Jensen’s medium^50^ ±100 μM cyclic-ERWGHDFIK; 200 μl was dispensed into each well and the chambered glass slides were placed in a static incubator set to 37 °C for 24 h, 48 h to allow biofilms to form. After biofilms were formed, the media was gently removed and the biofilms were washed with 200 μl PBS. The biofilms were then stained using Live/Dead BacLight Bacterial Viability Kits (Molecular Probes, Invitrogen) for 30 min, in the dark. The Kit uses SYTO 9 to label live cells (with intact cell membrane) and propidium iodide to label dead cells (with damaged cell membrane). The stained biofilms were then washed twice with PBS to remove excess stain. To assess the number of live/dead cells, stained biofilms were imaged using a Leica SP8 confocal microscope (Leica Microsystems) with a ×63 objective (HC PL APO 1.4 Oil). A 488 nm Argon laser was set to 4% power, and PMT detectors were set to a gain of 500 V. SYTO 9 was detected at 510–540 nm (green channel) and propidium iodide at 620–650 nm (magenta channel). Four biological replicates were imaged by collecting 10 *z* stacks (from bottom to top of biofilm; *z*-level interval 0.3 µm) from random areas of each well. BiofilmQ^51^ was used to segment images and quantify biofilm biomass (comparable to analysis with COMSTAT^52^). Bacterial cells were segmented in all *z* slices using manual thresholding to above the limit of background signal (that is, the average fluorescence intensity in unstained control samples (*n*_control_ = 10)). The used thresholds were 50 for the green channel and 40 for the magenta channel (8-bit gray values). Then, biofilm biomass (biofilm volume/biofilm substratum area (µm^3^/µm^2^)) was calculated for live cells (green channel) and dead cells (magenta channel). Then, the percentages of live biomass (green channel) and dead biomass (magenta channel) from total biomass (sum of green and magenta channel) were calculated. Data were tested for normal distribution and, when required, analyzed using a nonparametric test as indicated in the figure legends. A Kruskal–Wallis test was performed and there was no statistically significant difference between biological replicates. Images were analyzed in ImageJ^53^ and BiofilmQ^51^, and data were plotted using GraphPad Prism 9.3.

### Colony morphology assay

Cultures were grown overnight in LB medium and spotted in a 10-μl volume on solid media (1% tryptone, 1% agar, 20 μg ml^−1^ Coomassie blue and 40 μg ml^−1^ Congo red)^26^. Plates were incubated at room temperature for 6 d and then photographed with a Canon EOS 700D SLR camera fitted to a Leica MZ12 stereoscope.

### Growth curves

Growth curves were performed on a 96-well format. Overnight cultures grown in LB were diluted 1:40 into a final volume of 150 ml per well in the phosphate-buffered casein minimal medium, with arabinose added to a concentration of 25 mM where indicated, and the cyclic peptide added to a concentration of 10 μM or 100 μM as indicated. Four replicates of each condition/strain were performed. Absorbance measurements at 500 nm were made every 20 min for 18 h in a Tecan Spark plate reader, at 37 °C and with continuous shaking between measurements.

### dN/dS analysis of selective pressure on PaAP

For this analysis, we used the FUBAR analysis package^30^ to infer rates of synonymous (dS) and nonsynonymous (dN) mutation per amino acid position of PaAP, LasB and LasR. We used the 2,887 *P. aeruginosa* genomes part of the RefSeq database with an assembly level of at least ‘scaffold’ as of January 2023. After extracting *paaP*, *lasB* and *lasR* sequences from each using the command line BLAST tool^54^, sequences were filtered to remove those that had early termination codons or gapped alignments because the dN/dS analysis only analyzes point mutations. After this, 2,843 sequences for *paaP*, 2,858 sequences for *lasB* and 2,375 sequences for *lasR* were included in the analysis. These collapsed to 239 unique amino acid sequences for PaAP, 222 unique sequences for LasB and 459 unique sequences for LasR. Sequences were aligned using mafft^55^ and then the FUBAR package^30^ implemented through the datamonkey server^56^ was used to carry out the dN/dS analysis.

### Statistical analysis

All data are presented as mean ± standard error of the mean and were obtained from ≥3 independent experiments with total sample numbers provided in the figure legends. Statistical significance was evaluated with GraphPad Prism software, using a one-sample *t*-test. Significant difference assessed through an unpaired *t*-test, ****P < 0.0001.

### Reporting summary

Further information on research design is available in the Nature Portfolio Reporting Summary linked to this article.

## Online content

Any methods, additional references, Nature Portfolio reporting summaries, source data, extended data, supplementary information, acknowledgements, peer review information; details of author contributions and competing interests; and statements of data and code availability are available at 10.1038/s41589-023-01373-8.

## Supplementary information

Supplementary Information Supplementary Figs. 1–21, Note (purity and identification of peptides synthesized by Peptide Synthetics) and Tables 1–5.

Reporting Summary

Supplementary Video Conformational change PA domain undergoes from inhibited to active conformation.

## Data Availability

Structural data were deposited in the PDB and are available under accession numbers 8ACR, 8ACK, 8AC7, 8AC9 and 8ACG. Search components for molecular replacement comprised an ensemble of three PDBs previously deposited by others (6HC6, 5IB9 and 1TKJ). All other data are contained in the main paper and supplementary information. Raw data for figures are available for download on figshare (Fig. 1, 10.6084/m9.figshare.22766588; Fig. 2, 10.6084/m9.figshare.22766624; Fig. 3, 10.6084/m9.figshare.22766639 and Fig. 4, 10.6084/m9.figshare.22766705). All materials and reagents are available from the corresponding author. Source data are provided with this paper.

## Source data

Source Data Fig. 1: Statistical source data of Fig. 1d (mechanism of autoinhibition). Source Data Fig. 2: Statistical source data (kinetics of peptide degradation catalyzed by PaAP). Source Data Fig. 3: Statistical source data (structure-guided inhibitor design). Source Data Fig. 4: Statistical source data (PaAP in vivo assay).
