# Supplementary material for: An anti-biofilm cyclic peptide targets a secreted aminopeptidase from P. aeruginosa
Source: Nat Chem Biol. 2023 Jun 29;19(9):1158–66. doi: 10.1038/s41589-023-01373-8 (PMC10449631; doi:10.1038/s41589-023-01373-8)
Supplement: Supplementary file 1 — Supplementary Figs. 1–21, Note (purity and identification of peptides synthesized by Peptide Synthetics) and Tables 1–5. [file 41589_2023_1373_MOESM1_ESM.pdf]

nature chemical biology

Article

https://doi.org/10.1038/s41589-023-01373-8

An anti-biofilm cyclic peptide targets a secreted aminopeptidase from *P. aeruginosa*

In the format provided by the
authors and unedited

Table of Contents

Supplementary Figures **2**

Supplementary Figure 1. PaAP structural alignments 2

Supplementary Figure 2. Active site and Cation binding sites 3

Supplementary Figure 3. C-terminus H-bond network 3D 4

Supplementary Figure 4. C-terminus H-bond network 2D 5

Supplementary Figure 5. Crystal packing and C-term interaction with PA domain 6

Supplementary Figure 6. Superimposition of PaAP structures. 7

Supplementary Figure 7. Kinetic parameters of PaAP with aa-pNA substrates 8

Supplementary Figure 8. MALDI, PaAP peptide degradation activity 10

Supplementary Figure 9. LC-MS, PaAP peptide degradation activity 11

Supplementary Figure 10. Purity and Intact mass spec PaAP 12

Supplementary Figure 11. LC-MS, PaAP peptide degradation activity (Defensins) 14

Supplementary Figure 12. Peptide degradation assay 15

Supplementary Figure 13. Peptide degradation assay (full data plotted from Figure 2) 16

Supplementary Figure 14. Intact mass spec protein substrates +/- PaAP 17

Supplementary Figure 15. LC-MS, PaAP peptide degradation activity (cyclic-ERWGHDFIK) 18

Supplementary Figure 16. Inhibition of PaAP in function of time. 19

Supplementary Figure 17. Growth curves 20

Supplementary Figure 18. WT vs △PaAP preliminary biofilm assay 21

Supplementary Figure 19. Stability of cyclic-ERWGHDFIK 22

Supplementary Figure 20. FUBAR Analysis of PaAP, LasB, LasR 23

Supplementary Figure 21. Xray fluorescence scan data 24

Supplementary note on purity and identification of peptides synthesised by Peptide Synthetics **25**

Supplementary Tables **31**

Supplementary Table 1. Crystallographic Data Table 31

Supplementary Table 2. Crystallisation conditions 32

Supplementary Table 3. Primers 33

Supplementary Table 4. Strains 34

Supplementary Table 5. Fitted data 35

References **39**

Supplementary Figures

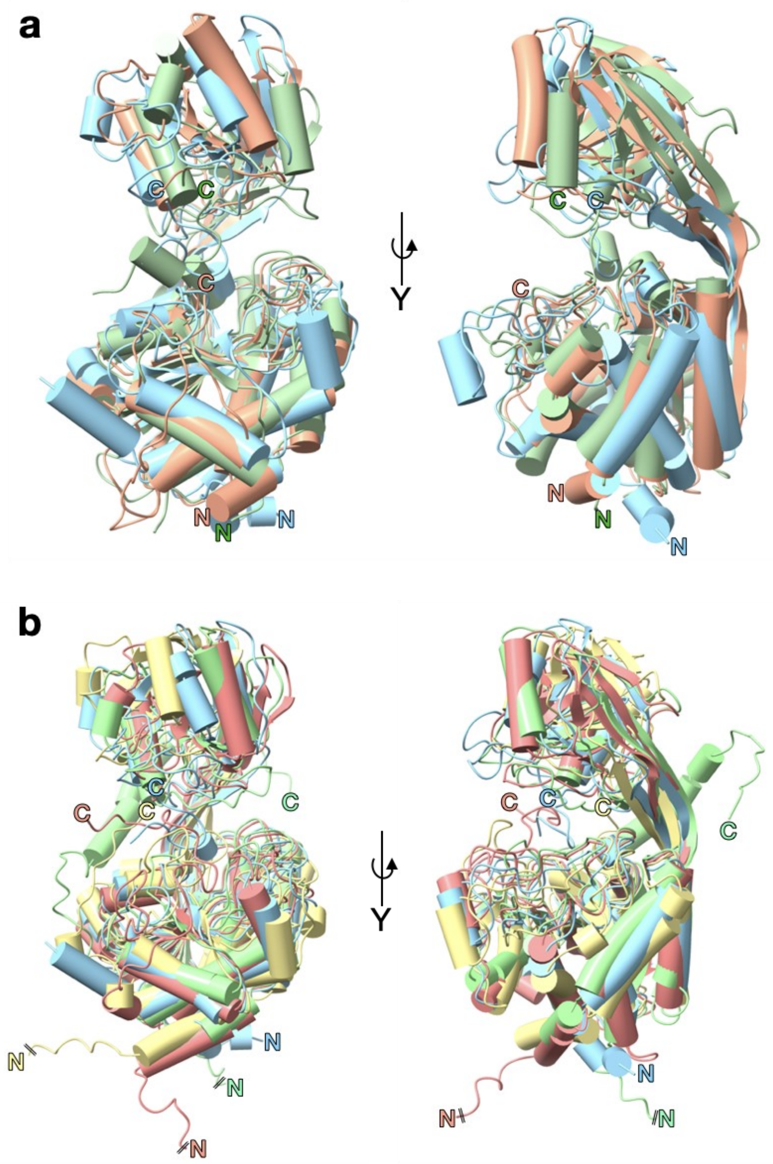

Supplementary Figure 1. PaAP structural alignments

a, Structural alignment of PaAP to homologues from PDB. The aminopeptidase and PA domains adopt similar conformations in these homologous structures. The C-terminus of PaAP and 6HC6 are bound in the cleft between the two domains. PaAP - blue, *Bacillus subtilis* (6HC6 - green) 2.98Å over 299 residues, *Aneurinibacillus sp*. AM-1 (2EK8 - coral) rmsd = 2.71Å over 357 residues. N & C-terminus labels are shown. Aligned by SSM secondary structure matching.

b, Structural alignment of PaAP to similar proteins from pathogenic organism in the Uniprot database. The Alpha fold predicted model was aligned with the structure of PaAP. The model confidence for the prediction of the N & C-terminal regions were low or very low for each homologue. The // shows the region the models were truncated too for illustration. PaAP - blue, *Fusarium oxysporum* (A0A0D2XET0 - green), *Streptomyces scabiei* (A0A086GRQ6 – coral), *Mycobacterium tuberculosis* (A0A051UGG7 – yellow).

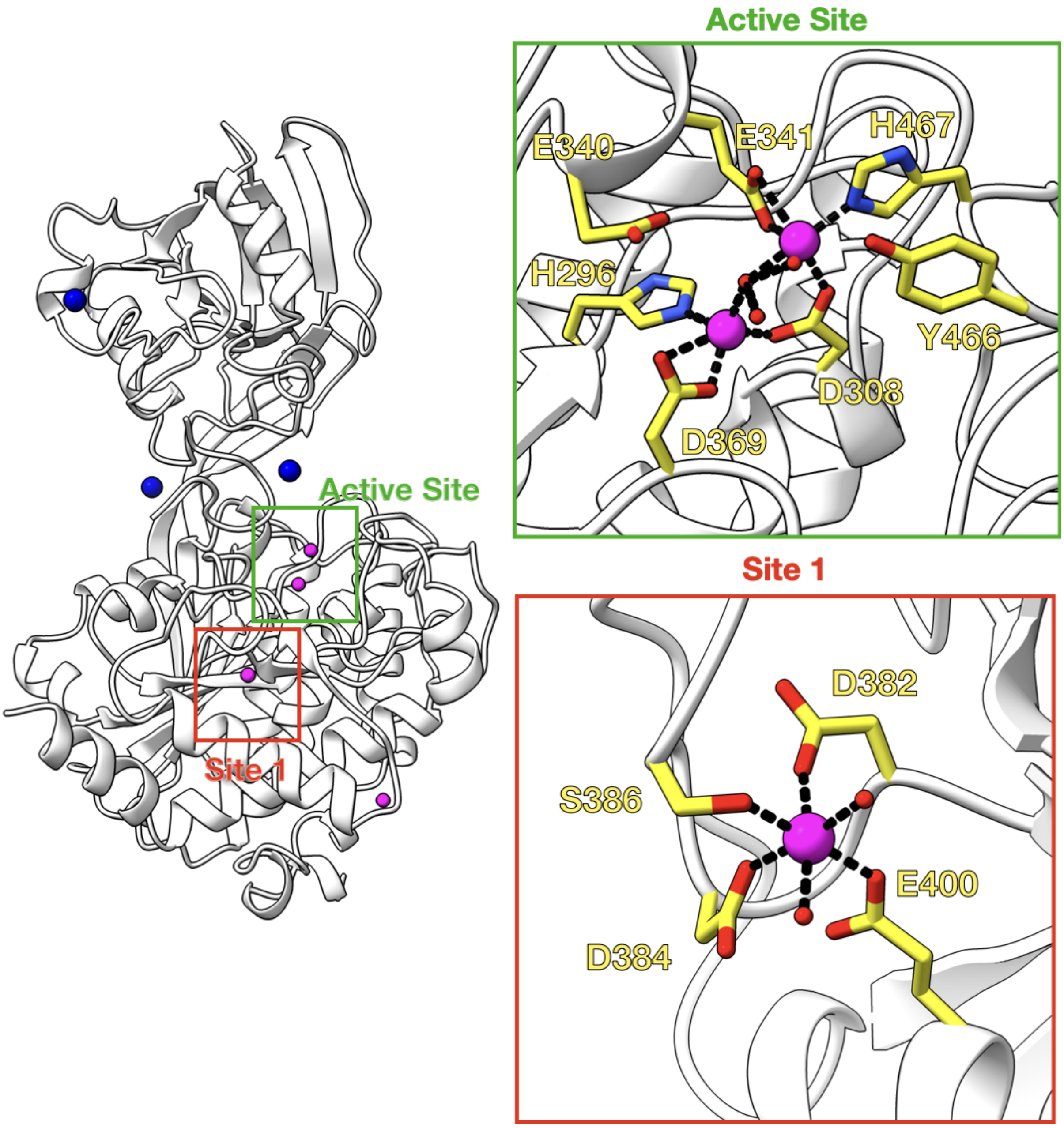

Supplementary Figure 2. Active site and Cation binding sites
The cation binding sites mapped onto the structure of full-length PaAP. Zinc ions shown as magenta spheres and sodium ions as blue spheres. The active site zinc ions are highlighted by the green box. The red box highlights site 1, zinc binding site.

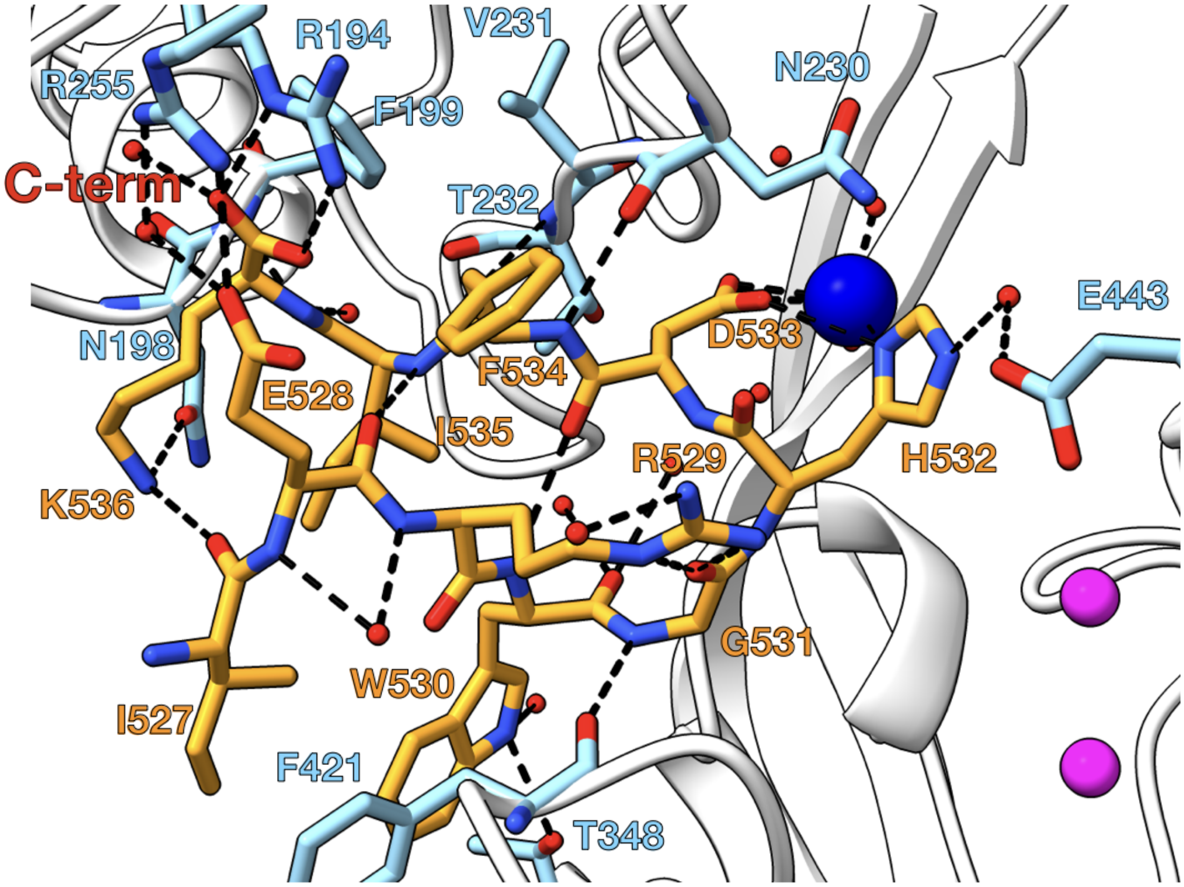

Supplementary Figure 3. C-terminus H-bond network 3D

The C-terminus of PaAP (residues 527-536) is shown in stick form with orange carbons. The residues of the main body of PaAP (PA and peptidase domains) are shown in with orange carbons. Sodium atom represented by a blue sphere, zinc atoms (highlighting the location of the active site) are represented as magenta spheres, and water molecules as red spheres. Hydrogen bonds are shown by black dashed lines. The C-terminus is labelled in red.

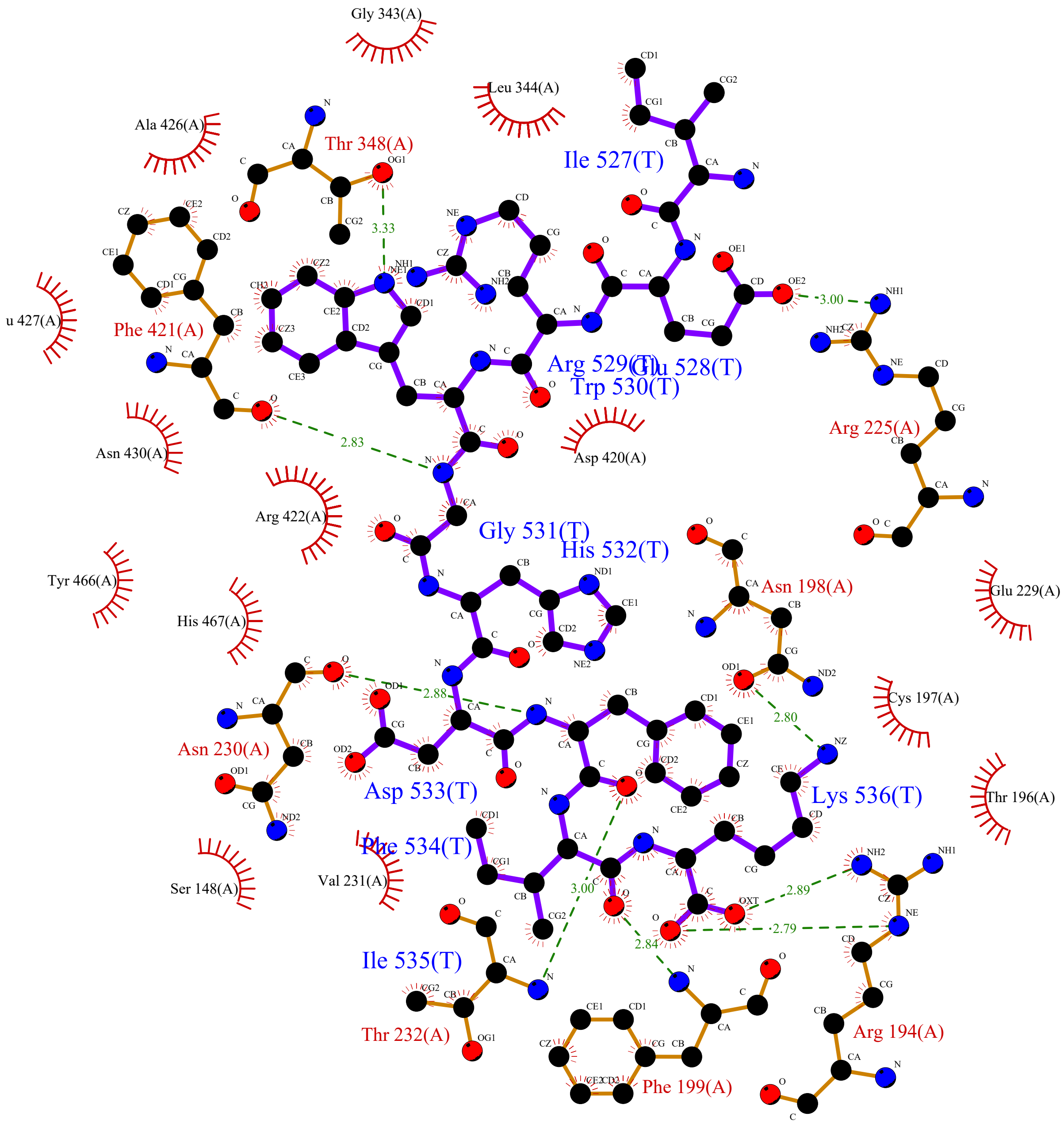

Supplementary Figure 4. C-terminus H-bond network 2D

Ligplot diagram showing the interaction the C-terminus of PaAP makes. C-terminus (purple bonds), PaAP maintain (orange bonds), hydrogen bonds green dashed lines, non-bonded contact shown by res semi-circles.

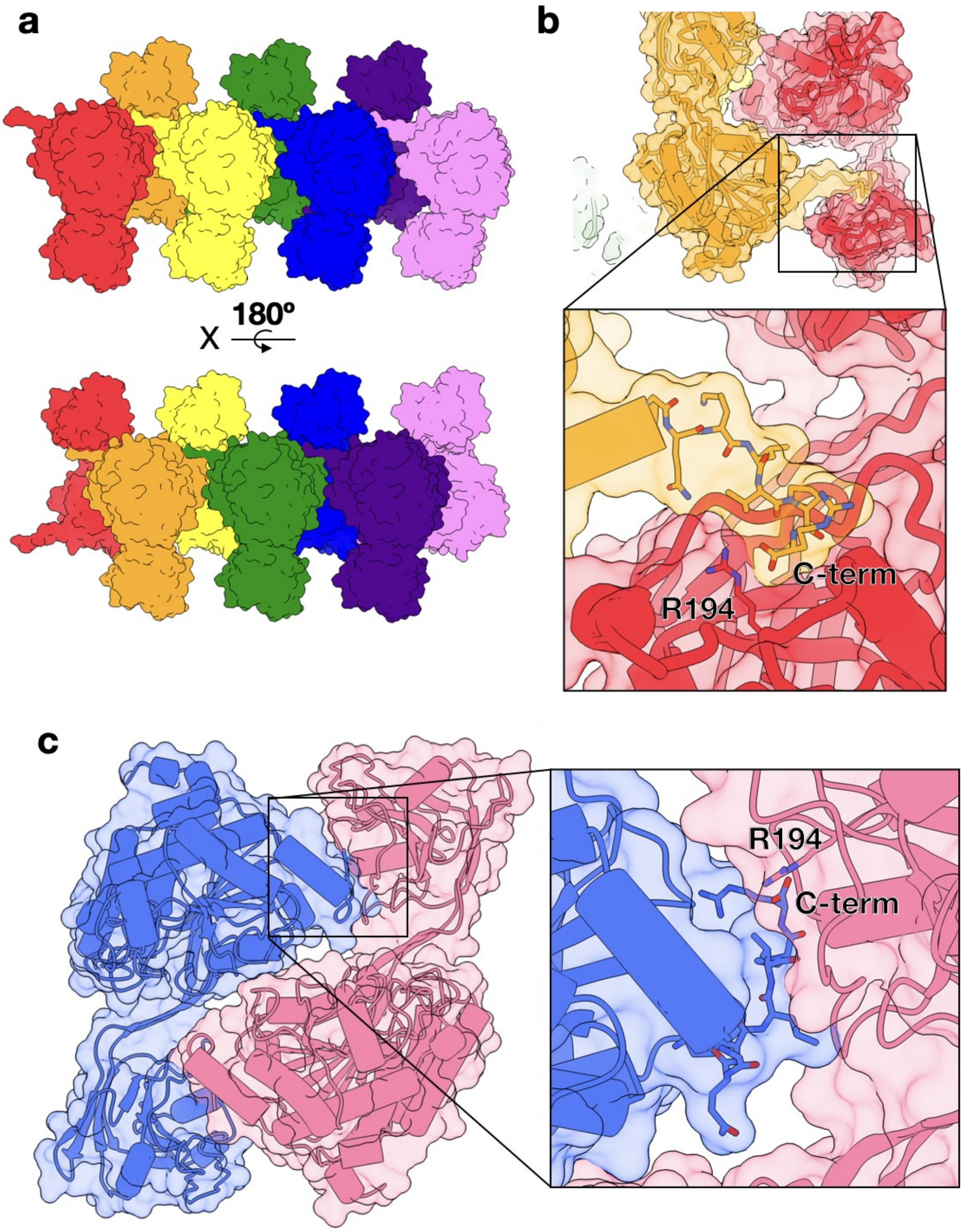

Supplementary Figure 5. Crystal packing and C-term interaction with PA domain
a PaAP_T crystallographic assembly. The protomers are linked in a chain via the C-terminus and PA domain. **b** Close up view of interaction between C-terminus and PA domain of adjacent protomers. **c** crystallographic dimer of $PaAP_{\triangle E340A(trunc)}$, a truncation mutant which did not require Thrombin cleavage. The C-terminus binds to the PA domain and interacts with the side chain of R194.

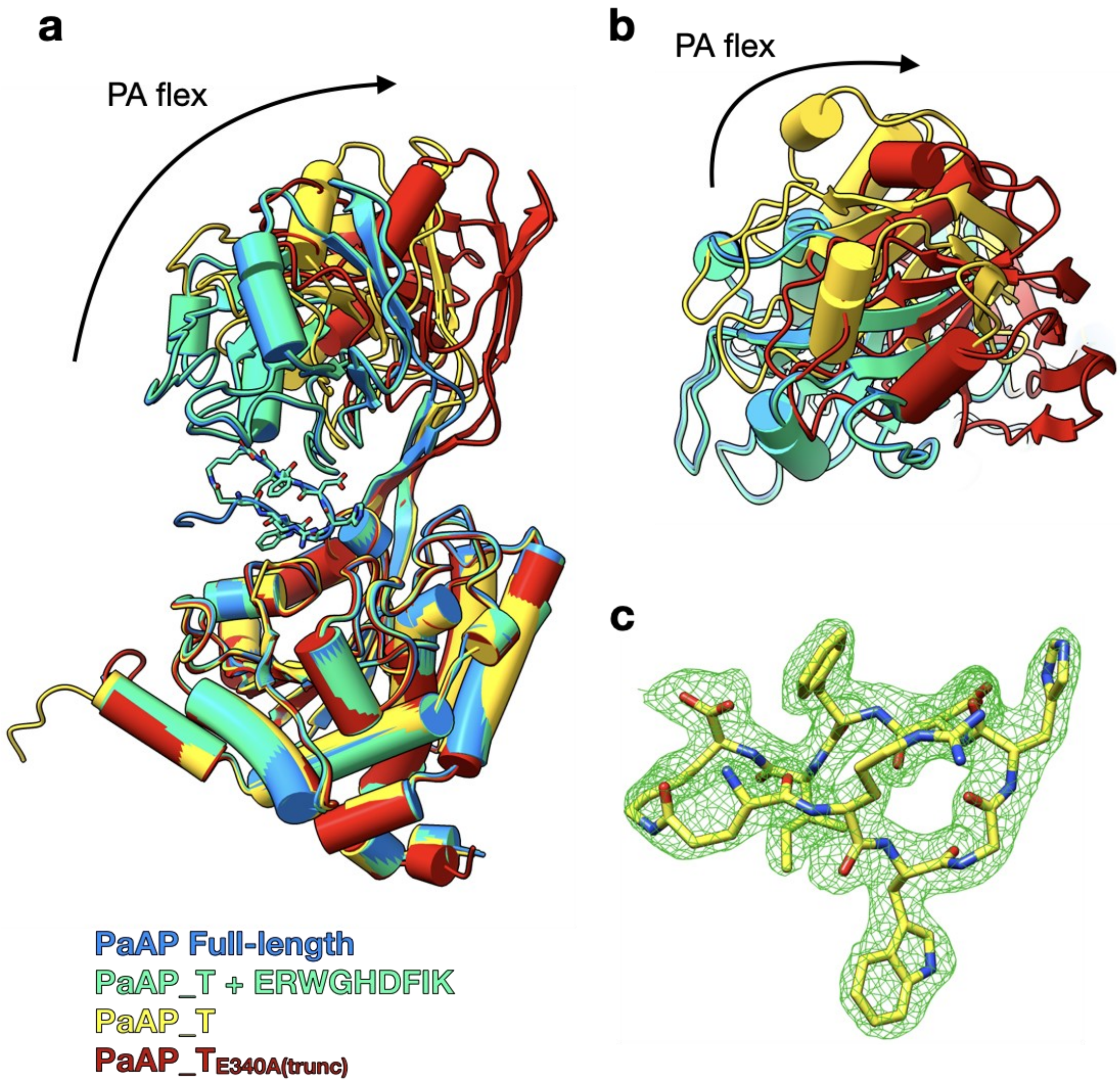

Supplementary Figure 6. Superimposition of PaAP structures.

a Comparison of PaAP structures, demonstrating the conformational change of the PA domain associated with an active and inhibited state. The Side view. **b** The top view. Superimposed by LSQ, maintains atoms of residues 274-510. The PA domain of PaAP_T undergoes a (~40° rotation) compared to the PA domain position in full-length PaAP structure. The PA domain in PaAP $_{\text{E340A(trunc)}}$, a truncation mutant not requiring thrombin cleavage (stop codon incorporated in thrombin cleavage site), adopts an alternative conformation. This suggests the PA domain is free to sample multiple conformations when not locked into place by interaction with the C-terminus. **c** OMIT map of ERWGHDFIK cyclic peptide, contoured at σ=2.5.

a

pNA Substrate	K_M (mM)	k_{cat} (min^{-1})	k_{cat} / K_M (mM^{-1} min^{-1})
Leu	2.29 +/- 0.14	689 +/- 22.1	301
Ile	1.29 +/- 0.16	11.2 +/- 0.53	8.68
Val	0.88 +/- 0.14	6.9 +/- 0.36	7.84
Ala	0.91 +/- 0.13	31.9 +/- 1.63	35.1
Met	1.95 +/- 0.07	57.5 +/- 4.81	29.4
Pro	1.68 +/- 0.15	29.6 +/- 1.13	17.6
Arg	0.91 +/- 0.08	67.2 +/- 1.97	73.8
Lys	1.53 +/- 0.19	248 +/- 12.9	162
Phe	1.1 +/- 0.25	2.34 +/- 0.26	2.12

b

Enzyme	K_M (mM)	k_{cat} (min^{-1})	k_{cat} / K_M (mM^{-1} min^{-1})
PaAP	0.61 +/- 0.02	6.14 +/- 0.08	10.1
PaAP_T	2.29 +/- 0.19	689 +/- 26.6	301
PaAP_T E340A	ND	ND	ND
PaAP_T R194A	3.55 +/- 0.25	516 +/- 19.5	145
PaAP_T Y466F	5.45 +/- 1.75	3.75 +/- 0.77	0.69

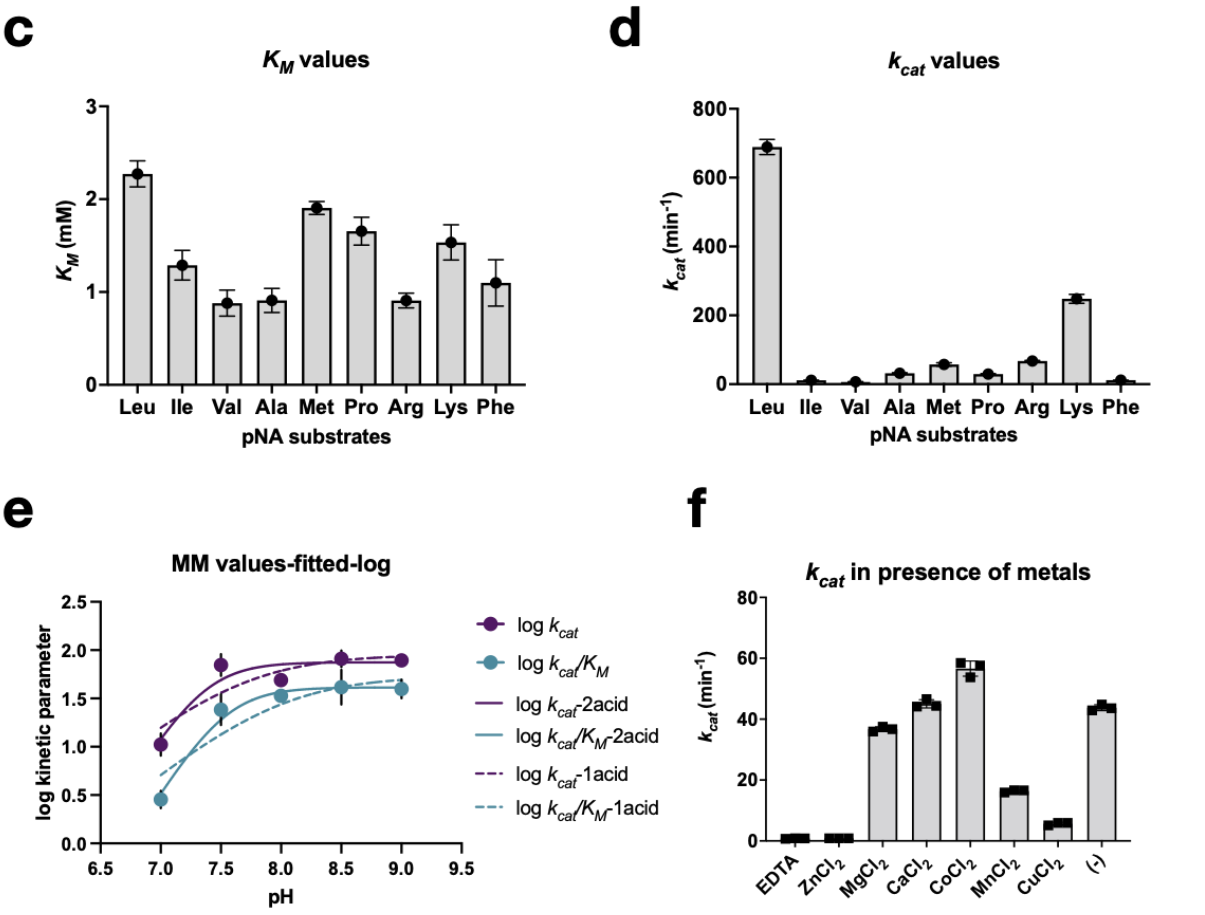

Supplementary Figure 7. Kinetic parameters of PaAP with aa-pNA substrates

a Table of kinetic parameters values for PaAP_T with different aa-pNA substrates. The +/- standard error is shown, from triplicate datapoints. **b** Table of kinetic parameters for WT PaAP and mutant forms. ND = no activity detected. No activity was detected for the E340A mutant. On the other hand, the Y466F mutant had a significantly reduced k_{cat} (3.75 min^{-1}) and increased K_M (5.45 mM), which is reflected in the k_{cat}/K_M being considerably lower than PaAP_T (0.69 $min^{-1}mM^{-1}$ compared to 301 $min^{-1}mM^{-1}$, respectively). These results support E340 to act as the general base, and Y466 to potentially stabilize the negative charge on the peptide tetrahedral intermediate by hydrogen bonding to the carboxylate oxygen atom [26, 27]. The activity of an additional mutant, R189A (located in the PA domain and interacting directly with the C-terminus of the full-length protein) was tested to investigate its role in catalysis. R189A had comparable k_{cat} (516 min^{-1}) and K_M (3.55 mM) values to PaAP_T

suggesting that, at least in terms of the minimal aa-pNA substrate, it is not involved in substrate binding and/or catalysis. **c** Bar graph plotting K_M values of different pNA substrates. **d** Bar graph plotting k_{cat} values of different pNA substrates. **e** Plot of k_{cat} values at different pH. Enzyme activity was optimal at pH values > 8.0, with the activity decreasing drastically below neutral pH, in a profile akin to other zinc-dependant metalloproteases [25]. Data fitting yielded two ionizable groups with an apparent pK_a of approximately 7.5 both on k_{cat} and k_{cat}/K_M. Because this pK_a value is likely too low to be the substrate N-terminal amine, this suggests deprotonated active site residues are required for substrate binding and catalysis, in keeping with the generally accepted catalytic mechanism which requires an active site glutamate to act as a general base and deprotonate a water molecule coordinated by the two active site zinc molecules[1]. **f** Graph of k_{cat} values ion the presence of different divalent metals. PaAP was active in a standard assay buffer, with no metal ion supplement, suggesting catalytic metal ions were co-purified. Structure determination by X-ray crystallography and X-ray fluorescence spectrum of crystals unambiguously confirmed two zinc ions are bound in the active site (Supplementary Figs. 2 and 21). Regardless, we were interested in the catalytic activity of PaAP in the presence of alternative divalent metal ions. Surprisingly, excess zinc reduced catalytic activity to a similar degree as EDTA treatment. One explanation for this could be the presence of allosteric zinc ion binding sites, which were determined in the crystal structure. One of the binding sites (site 1, residues D382, D384, S386 and E400) strongly coordinates a zinc ion in the different crystal forms (Supplementary Fig. 2). This allosteric zinc site may be inhibitory at higher concentrations of zinc. For c-f, mean and standard error shown for datapoints measured in triplicate independent measurements.

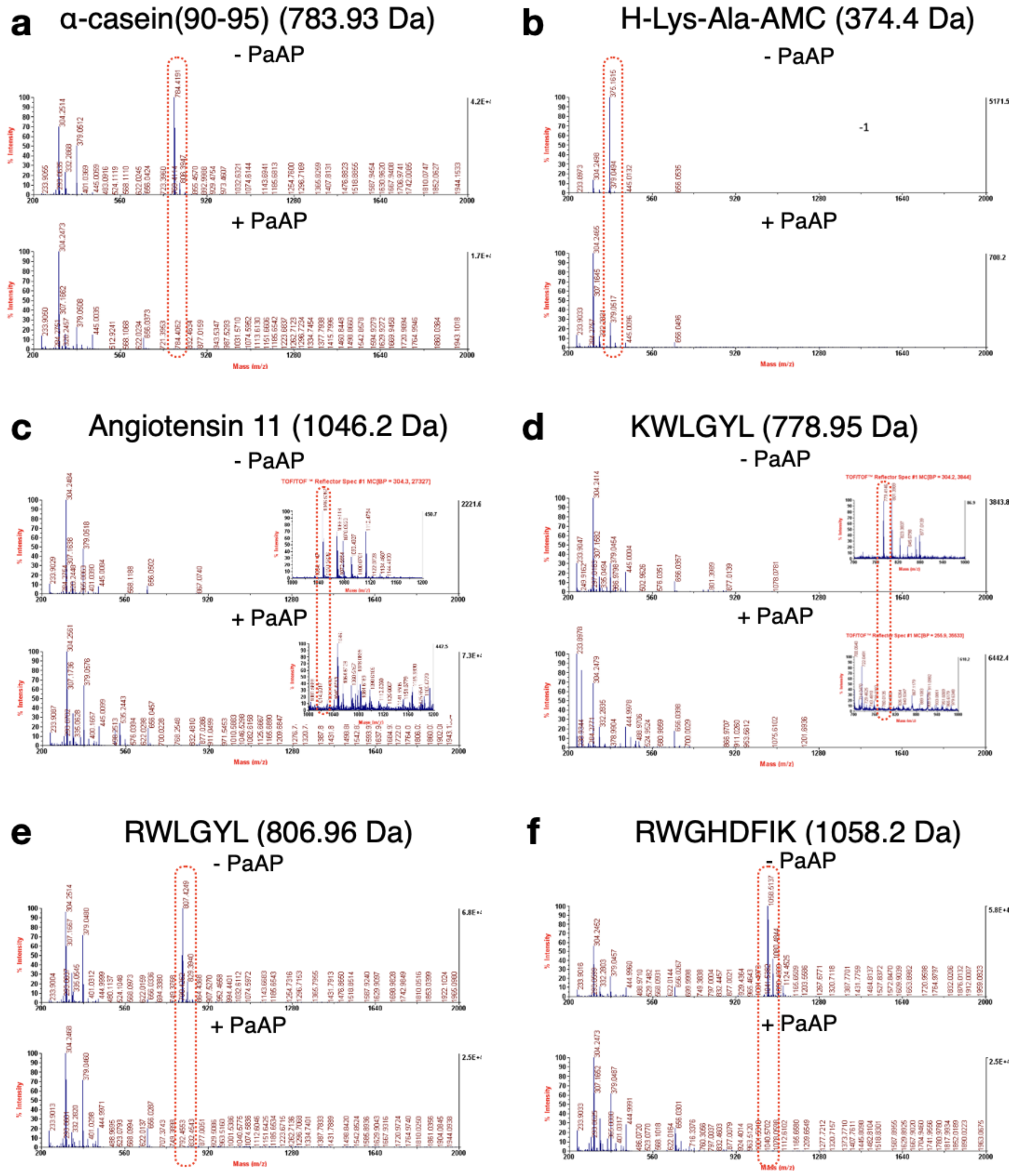

Supplementary Figure 8. MALDI, PaAP peptide degradation activity

Six peptides were incubated in the presence of PaAP, alongside a control (PaAP excluded). The samples were then analysed by MALDI. Peaks corresponding the the mass of the peptides were present in the control samples. However, these masses were absent in the samples incubated with PaAP, suggesting the peptides had been degraded.

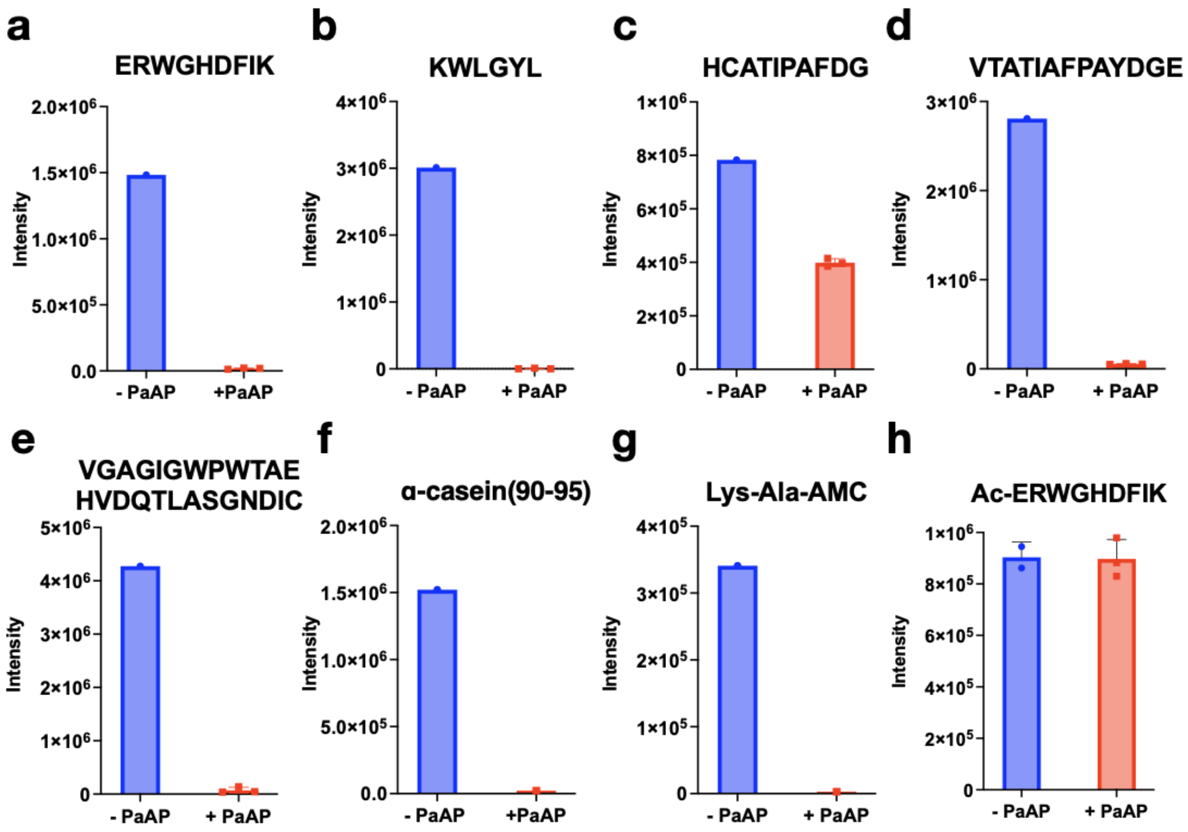

Supplementary Figure 9. LC-MS, PaAP peptide degradation activity

a-h, different peptides (starting sequence indicated on the top of each panel) were incubated in the presence of absence of PaAP. The graphs show the peak area for the peptide mass in the absence of PaAP (blue) and the presence of PaAP (red). The reduction in peak area is evidence of PaAPs peptidase activity. Moreover, mass searches identified breakdown products for these peptide substrates. The assays were performed in triplicate (a,b,c,d,e,h) or single samples for those already investigated by MALDI (f,g). Bar height represents the mean and error bars show the standard deviation.

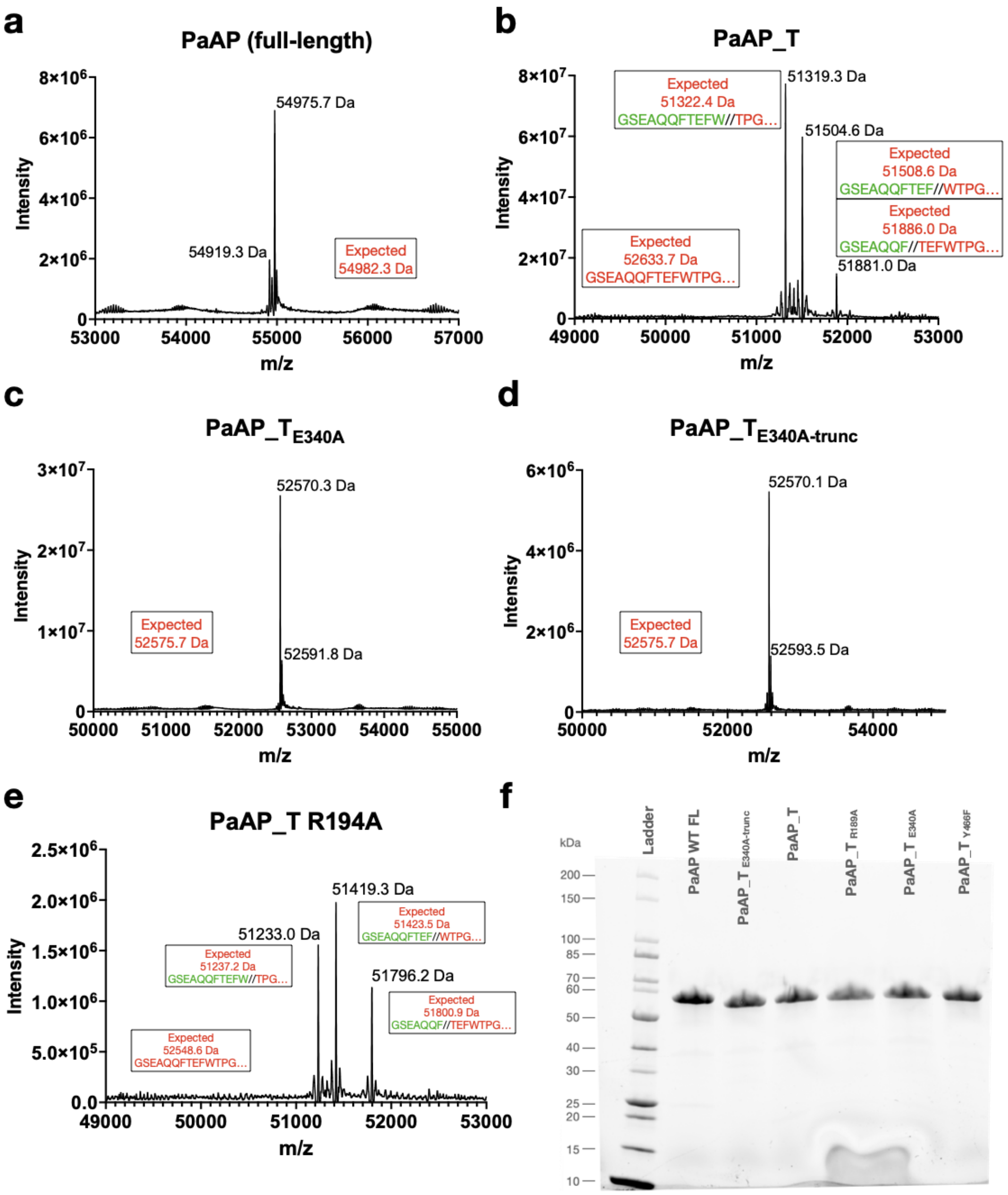

Supplementary Figure 10. Purity and Intact mass spec PaAP

a Intact mass of full-length PaAP. **b** Intact mass of PaAP_T (thrombin cleaved). The mass was considerably different than expected. The mass difference is due to auto-processing of N-terminus. The expected mass of cleaved products agreed with detected masses. The first 14aa from the N-terminus of PaAP are shown in red. The cleavage site is represented by //. The amino acids in green are removed. Intact mass spec of PaAP_T had three mass peaks (m/z = 51881.0, 51504.6 & 51319.3), corresponding to species starting with TEFWTPGKP, WTPGKP and TPGKP respectively (removal of 7, 10 & 11 residues from N-terminus (-GSEAQQF, - GSEAQQFTEF & - GSEAQQFTEFW, respectively) and the same thrombin cleaved C-terminus (ending VPR). **c** Intact mass of PaAP_T_{E340A}. Inactive PaAP_T_{E340A} had a single peak (m/z = 52570.2), corresponding to a species with a thrombin cleaved C-terminus (ending VPR) and a TEV cleaved N-terminus (starting GSE), with a E340A mutation. Interesting this construct co-purified and co-crystallised with the C-terminus bound in the groove between the PA domain and peptidase domain (PDB: 8ACR, unambiguous density, similar to FL structure). This suggests in active PaAP_T, once the C-term has been cleaved, the enzyme degrades the peptide. **d** Intact mass of PaAP_$T_{E340A\text{-}trunc}$ (truncation via stop codon, no thrombin cleavage step required). **e** Intact mass of PaAP_T_{R194A}. The mass

was considerably different to expected, this is due to auto-processing of N-terminus similarly to in (b). All data processed to 0.1Da using MaxEnt 1. All mass values for PaAP are approximately -5 Da from their expected mass. Intact mass spec of purified PaAP was performed once (n=1). **f** SDS-PAGE depicting purified PaAP variants studied. FL, full length; _T, truncated. PaAP was purified on several occasions with a similar degree of purity and characteristics.

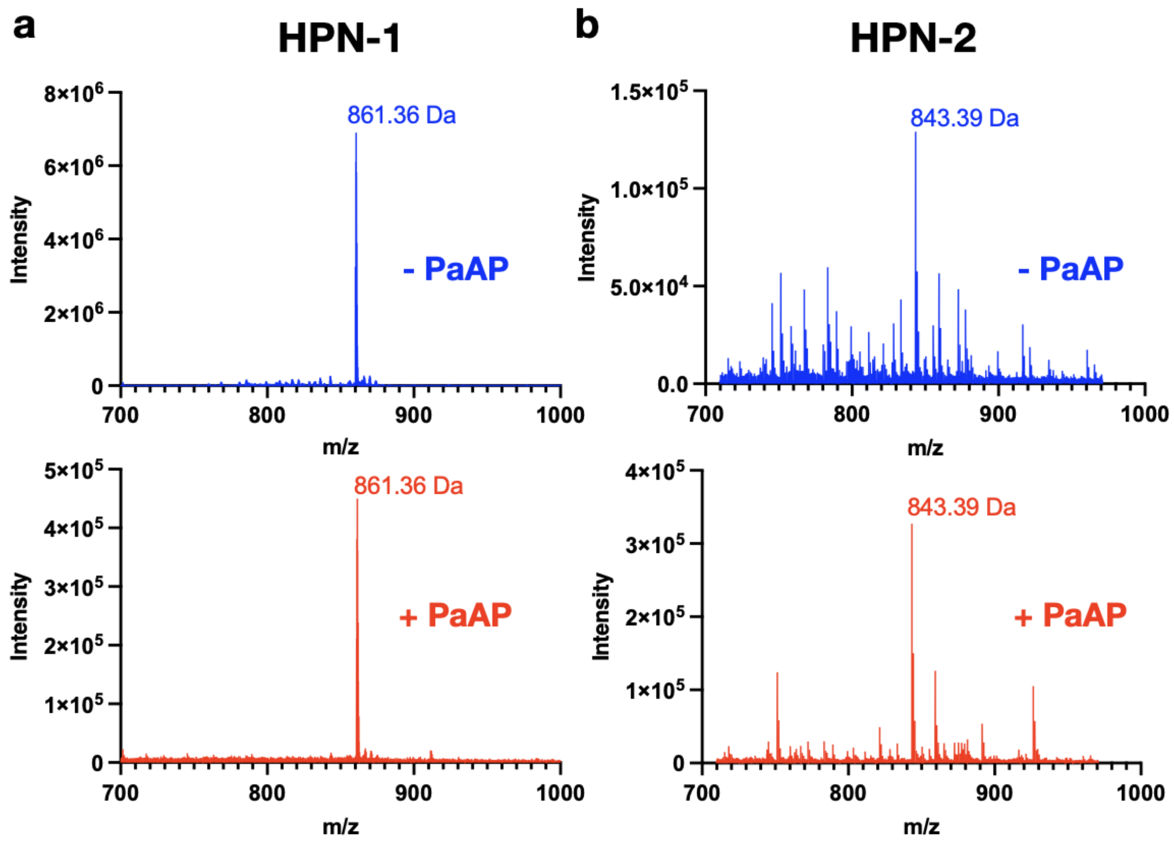

Supplementary Figure 11. LC-MS, PaAP peptide degradation activity (Defensins)
Human defensins HPN-1 (**A**) and HPN-2 (**B**) were incubated in the presence (red) and absence (blue) of PaAP. The graphs show the mass peak for HPN-1 and HPN-2. Note the mass peak is present in both + and - PaAP samples suggesting no degradation occurred. Mass searches were performed to identify breakdown products, which did not return any peaks in the chromatogram.

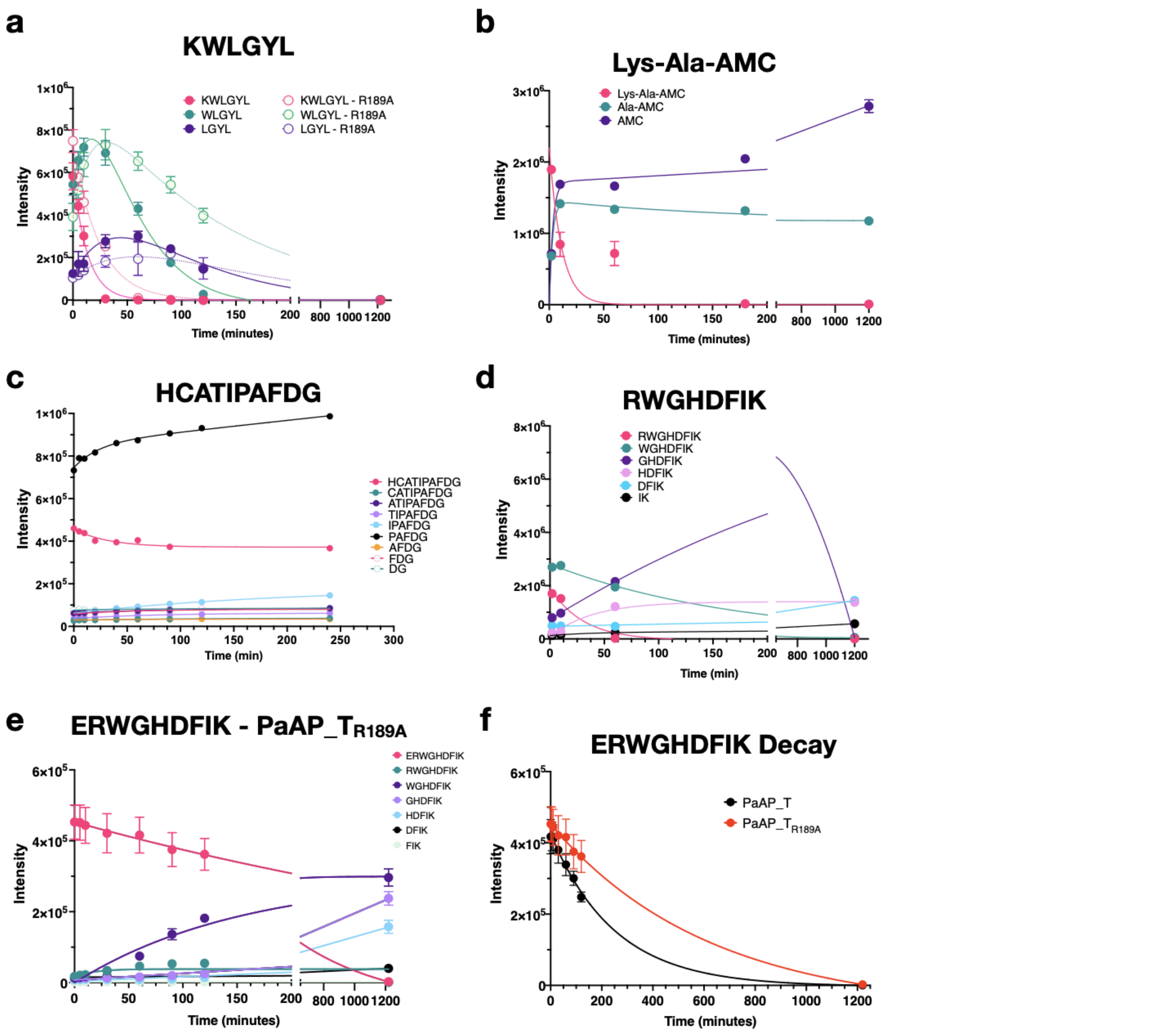

Supplementary Figure 12. Peptide degradation assay

Peptides were incubated in the presence of PaAP. LC-MS was used to detect peptide fragments of samples quenched at different timepoints. Each datapoint was measured in triplicate and the data was fit to XXX using prism. **a** The peptide KWLGYL was incubated with either PaAP_T (solid fill circles) or PaAP_T$_{R189A}$ (no fill circles). The R189A mutant degrades peptide at a slower rate. **b** The peptide Lys-ALa-AMC incubated with either PaAP_T. **c** The peptide HCATIPAFDG incubated with either PaAP_T. **d** The peptide RWGHDFIK incubated with either PaAP_T. **e** The peptide ERWGHDFIK incubated with either PaAP_T$_{R189A}$. **f** Comparison between decay of ERWGHDFIK peptide when incubated with PaAP_T or PaAP_T$_{R189A}$. Mean and standard error shown for datapoints measured in triplicate independent measurements.

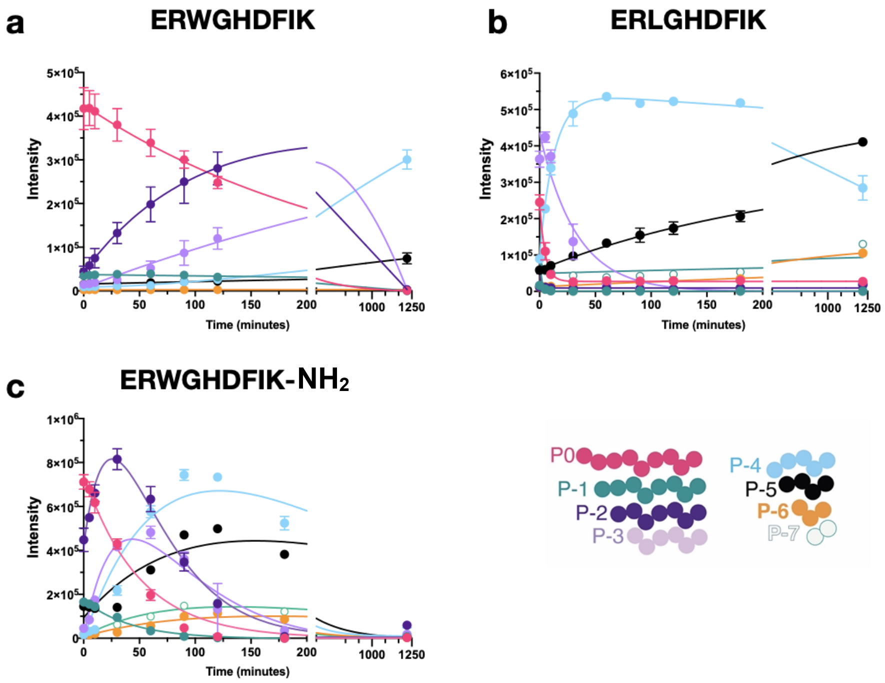

Supplementary Figure 13. Peptide degradation assay (full data plotted from Figure 2)
The full data from figure 2 is plotted in these graphs. **a** The peptide ERWGHDIK was incubated with PaAP_T. **b** The peptide ERLGHDFIK was incubated with either PaAP_T. **c** The peptide ERWGHDFIK-NH_2 was incubated with ePaAP_T. The key is shown in the bottom right panel. Mean and standard error shown for datapoints measured in triplicate independent measurements.

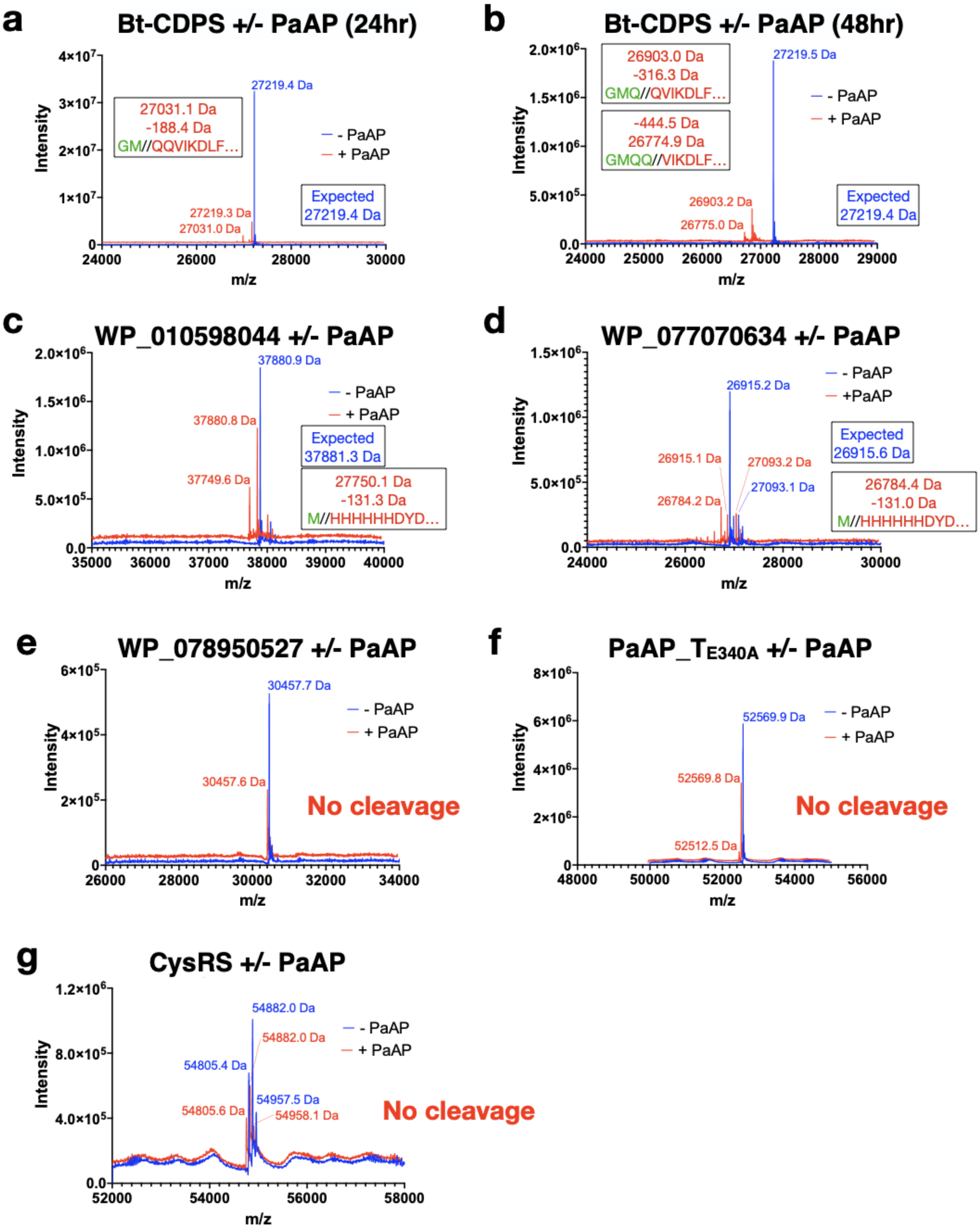

Supplementary Figure 14. Intact mass spec protein substrates +/- PaAP

The intact mass was determined for 6 randomly selected proteins after incubation with PaAP (+, red trace). A control, where PaAP was excluded (-, blue trace). The control trace (-, red) has been offset (x = -50, y + (10000 to 100000 depending on plot)) to aid visualising the data. The mass shifts were calculated, which corresponds to loss of N-terminal amino acids, which are displayed on the plots (**a-g**). Samples with a detectable mass difference were assumed to have been caused by protease activity. The mass shifts were calculated and shown in red. The liberated N-terminal amino acids are shown in green and the cleave site highlighted by //.

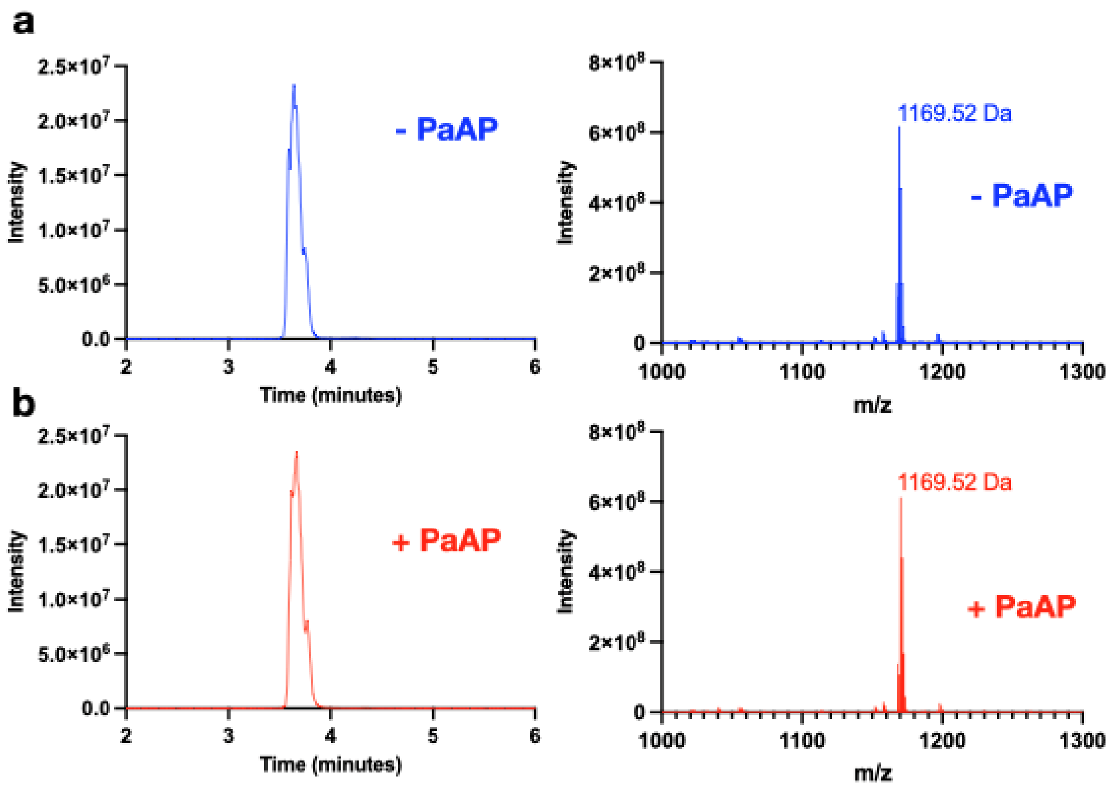

Supplementary Figure 15. LC-MS, PaAP peptide degradation activity (cyclic-ERWGHDFIK)

Cyclic-ERWGHDFIK were incubated in the presence (red) and absence (blue) of PaAP. The graphs show the TIC chromatogram (left) and the m/z plot (right). Cyclic-ERWGHDFIK was detected to similar levels in both +/- samples and not peptide break down products were identified, which suggests PaAP is unable to degrade cyclic-ERWGHDFIK.

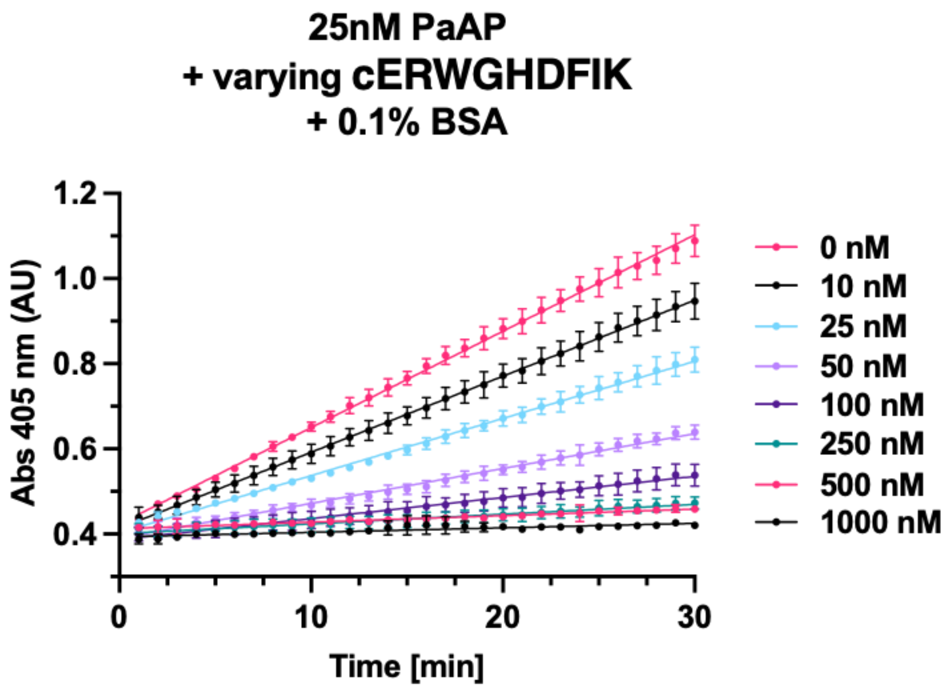

Supplementary Figure 16. Inhibition of PaAP in function of time. Raw data showing an assay with 25nM PaAP and increasing concentration of cyclic-ERWGHDFIK peptide. Over 30 minutes no curvature on the inhibited curves in comparison to uninhibited (pink) was observed, which is diagnostic of no time-dependent inhibition. 0.1% BSA (bovine serum albumin) was included to stabilize PaAP over the reaction time. Mean and standard error shown for datapoints measured in triplicate independent measurements.

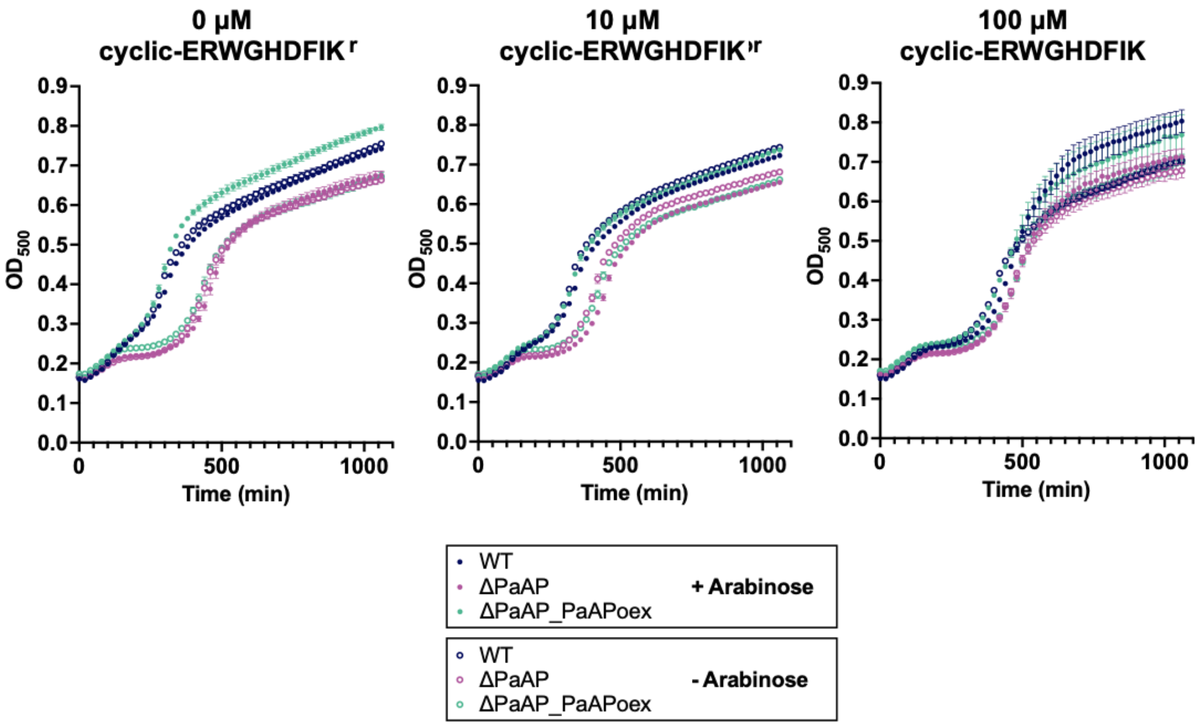

Supplementary Figure 17. Growth curves

The growth of Pseudomonas aeruginosa was monitored by measuring the absorbance at OD_{500}. Growth was assayed in casein media. Three strains were tested WT, ΔPaAP (PaAp deletion mutant) and ΔPaAP_PaAPoex (the only copy of PaAP under the control of an arabinose-inducible P_{BAD} promoter). The panels left to right shows the growth in the presence of an increased concentration of cyclic-ERWGHDFIK (0, 10 and 100 µM). Datapoints show the mean of four independent measurements (n=4) and standard error.

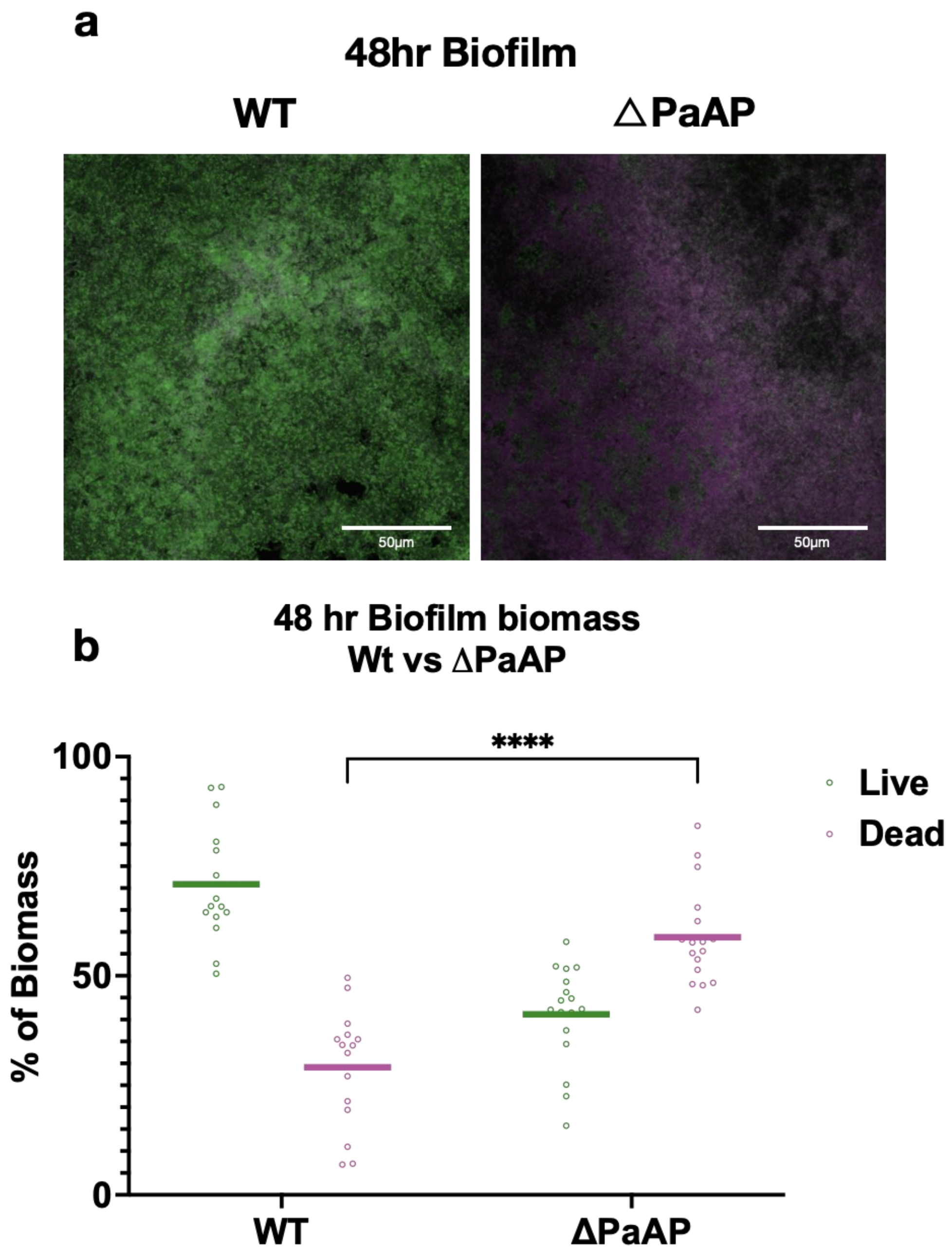

Supplementary Figure 18. WT vs △PaAP preliminary biofilm assay

a, Representative images of 48 hour old WT and △PaAP biofilms, which had been stained with LIVE/ DEAD® BacLight™. **b,** The percentage of biofilm biomass for green (live cells) and magenta (dead cells) channels is plotted for WT and △PaAP. Significant difference assessed through a two-sided unpaired t-test, **** p-<0.000001. N = 3 biological replicates with 5 random *z*-stack images (15 data-points). Mean of replicates is represented by a bar.

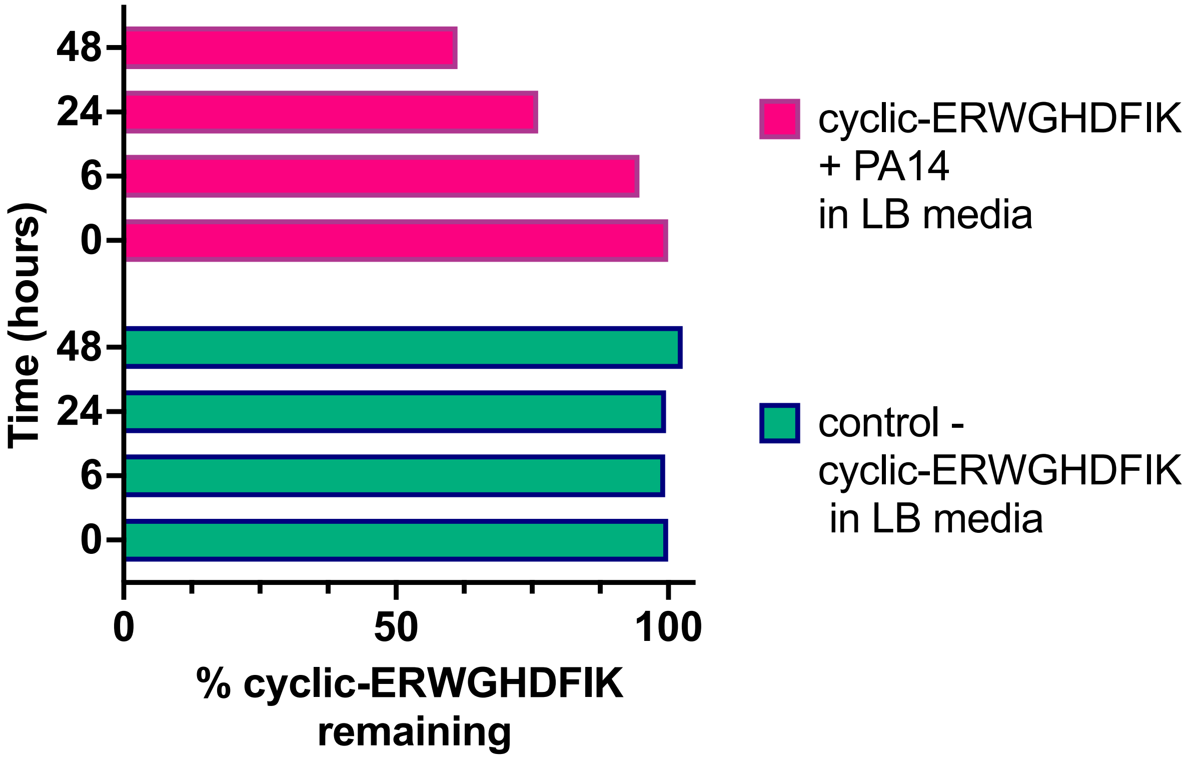

Supplementary Figure 19. Stability of cyclic-ERWGHDFIK

The stability of cyclic-ERWGHDFIK was tested by incubating in LB media +/- inoculation with PA14 Pseudomonas aeruginosa. Samples were taken at set timepoints over a 48 hour time period, quenched and analysed by LC-MS. % of cyclic-ERWGHDFIK remaining was calculated by comparing integrated peak areas to the 0 time point. n=1

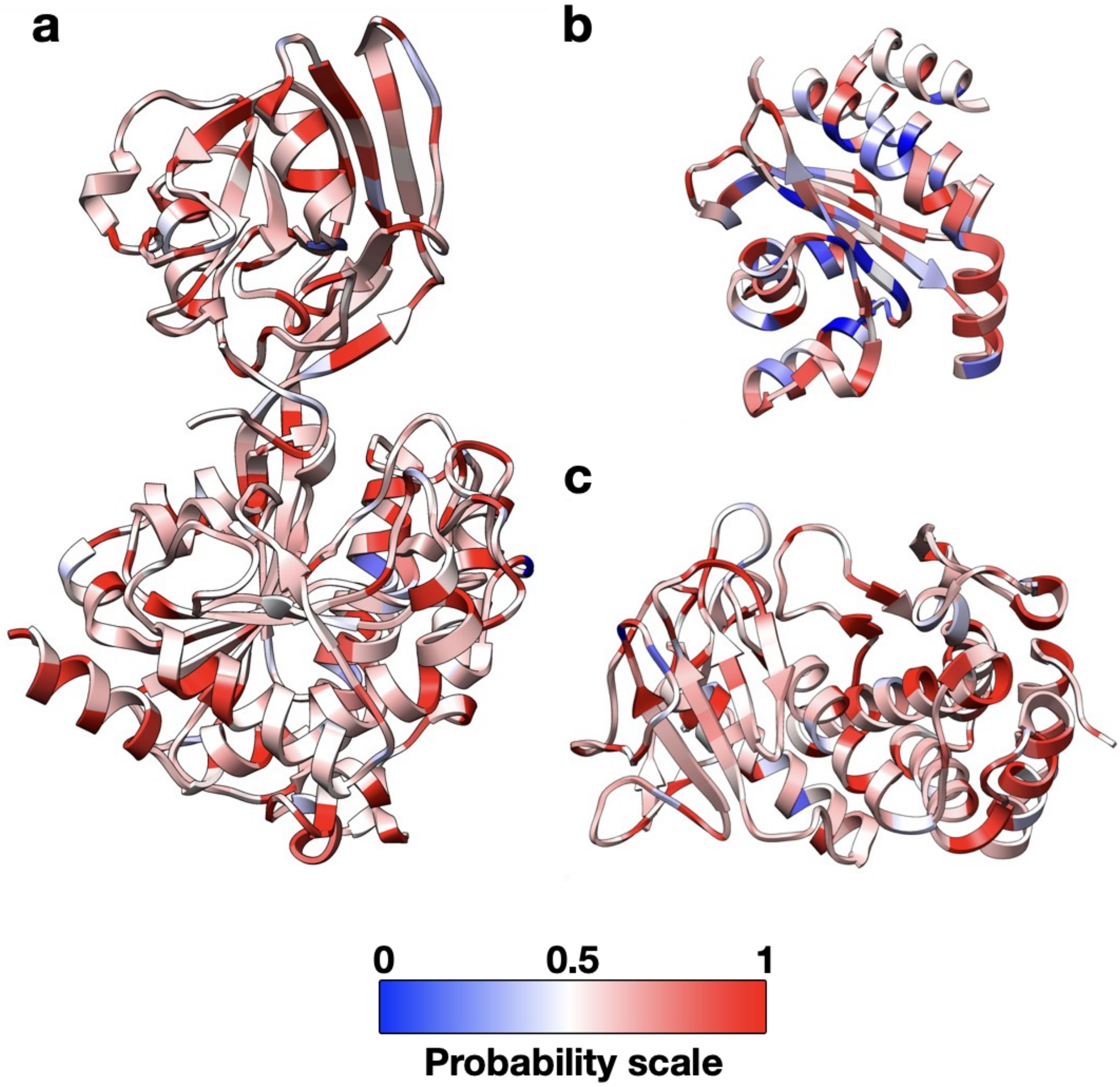

Supplementary Figure 20. FUBAR Analysis of PaAP, LasB, LasR

FUBAR was used to infer rates of synonymous and non-synonymous mutations at each amino acid position. Synonymous substitution rates (α) are assumed neutral and and nonsynonymous substitution rates (β) indicate diversifying selection if they are higher or purifying selection if they are lower. Bayesian approximation was used to calculate the posterior probability of negative (purifying) selection (α > β) for each site from 1 (red, synonymous mutation rate certain to be greater than non-synonymous mutation rate) to 0 (blue, synonymous mutation rate certain to be lower than non-synonymous mutation rate). Colours for probability of negative selection were mapped onto the structure of PaAP (a), LasR (b) and LasB (c).

A

Zn edge scan

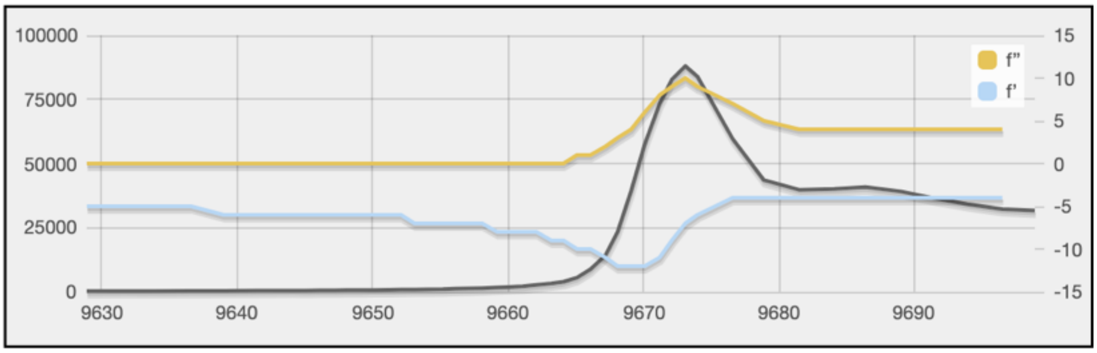

E(Peak): 9673.1 eV (1.2817 Å)	f”: 10.11 / f’ -7.91 e
E(Inf): 9669.1 eV (1.2823 Å)	f”: 4.81 / f’: -12.93 e
Exposure: 1.00 s	Transmission: 1.00 %
Beamsize: 30x30 µm	Axis position: 0.00 °

B

MCA spectra

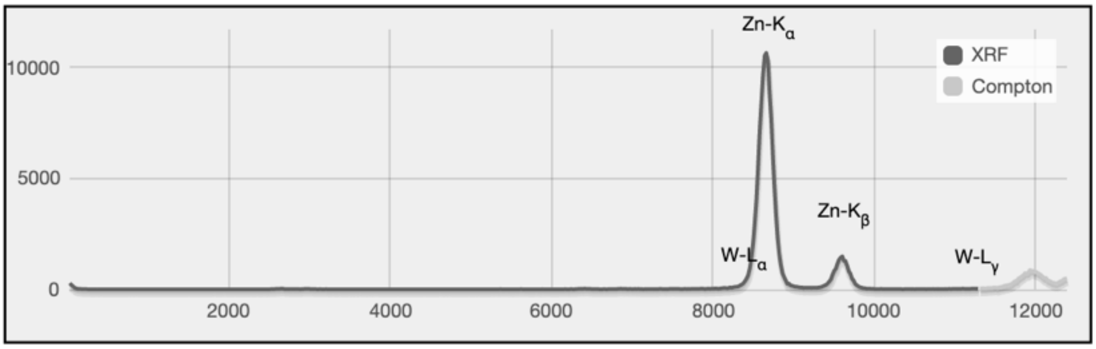

Exposure: 1.00 s	Energy: 12399.6 eV
Beamsize: 30x30 µm	Transmission: 8.00 %

Supplementary Figure 21. Xray fluorescence scan data

A Zn edge scan of WT PaAP crystal. **B** MCA spectra of WT PaAP crystal.

Supplementary note on purity and identification of peptides synthesised by Peptide Synthetics

ERWGHDFIK

a

University of St Andrews
Customer report: 62653 — Date :23rd November 2021

Compound code	
Sequence	ERWGHDFIK
N-terminus	Amine
C-terminus	Acid
Other modifications	None
Other/comments	
Counter Ion (if present):	TFA
Molecular mass (Av)	1187.304
Amount	5.0mg
Batch no	01
Purity (Determined by HPLC - see enc)	>90%
Physical properties	Lyophilised off-white powdered solid
Storage conditions	Store lyophilised at ≤ -20°C

b

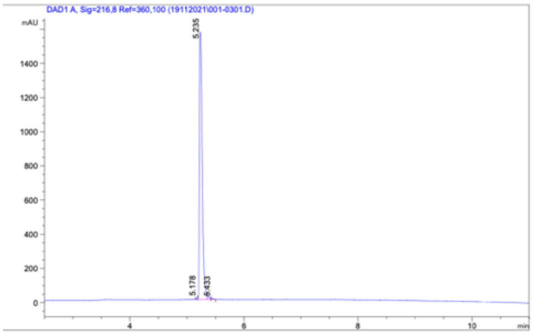

c

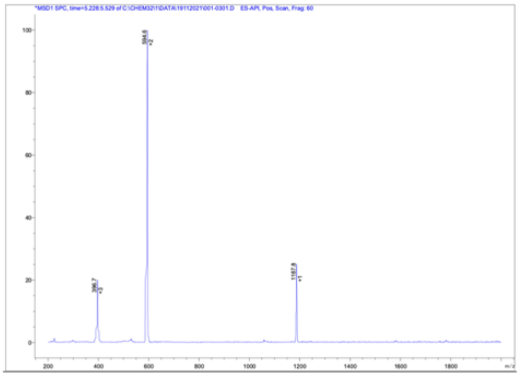

#	Meas. Ret.	Area	Area %
1	5.178	16.536	0.307
2	5.235	5302.439	98.341
3	5.353	56.711	1.052
4	5.433	16.189	0.300

Purity and identification of peptides synthesised by Peptide Synthetics – ERWGHDFIK
a Peptide synthetics report summary. **b** HPLC trace. **c** Mass Spectrometry trace.

ERWGHDFIK
cyclised head-to-tail

a

University of St Andrews
Customer report: 64880

Date :28th June 2022

Compound code	
Sequence	(ERWGHDFIK)
N-terminus	N/A
C-terminus	N/A
Other modifications	Cyclized head to tail
Other/comments	
Counter Ion (if present):	TFA
Molecular mass (Av)	1169.289
Amount	1.0mg
Batch no	01
Purity (Determined by HPLC - see enc)	>90%
Physical properties	Lyophilised off-white powdered solid
Storage conditions	Store lyophilised at ≤ -20˚C

b

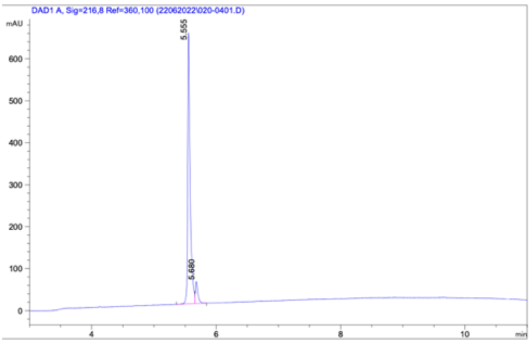

#	Meas. Ret.	Area	Area %
1	5.555	1724.043	92.378
2	5.680	142.245	7.622

c

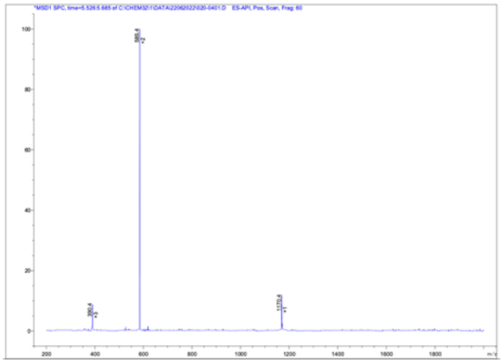

Purity and identification of peptides synthesised by Peptide Synthetics – ERWGHDFIK cyclised head-to-tail

a Peptide synthetics report summary. **b** HPLC trace. **c** Mass Spectrometry trace.

cyclic-ERWGHDFIK
cyclised isopeptide bond

a

University of St Andrews
Customer report: 66931

Date :9th November 2022

Compound code	
Sequence	(ERWGHDFIK) Cyclised via side chains of E and K
N-terminus	Amine
C-terminus	Acid
Other modifications	None
Other/comments	
Counter Ion (if present):	TFA
Molecular mass (Av)	1169.289
Amount	5.0mg (5 x 1.0mg)
Batch no	01
Purity (Determined by HPLC - see enc)	>90%
Physical properties	Lyophilised off-white powdered solid
Storage conditions	Store lyophilised at ≤ -20˚C

b

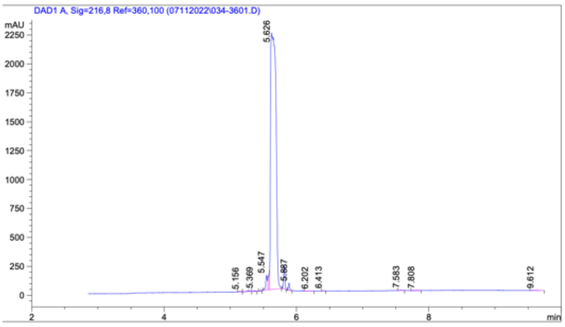

c

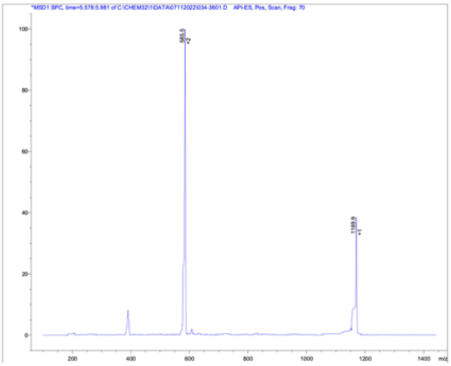

#	Meas. Ret.	Area	Area %
1	5.156	8.042	0.061
2	5.278	24.598	0.186
3	5.369	11.430	0.087
4	5.460	6.025	0.046
5	5.547	230.184	1.745
6	5.588	186.828	1.416
7	5.626	12358.707	93.677
8	5.816	254.848	1.932
9	5.887	74.525	0.565
10	6.202	5.491	0.042
11	6.413	9.032	0.068
12	7.583	10.439	0.079
13	7.808	5.614	0.043
14	9.612	7.070	0.054

Purity and identification of peptides synthesised by Peptide Synthetics – cyclic-ERWGHDFIK cyclised isopeptide bond

a Peptide synthetics report summary. **b** HPLC trace. **c** Mass Spectrometry trace.

Ac-ERWGHDFIK

a

University of St Andrews
Customer report: 69027 Date :10th March 2023

Compound code	
Sequence	Ac-ERWGHDFIK
N-terminus	Acetyl
C-terminus	Acid
Other modifications	None
Other/comments	
Counter Ion (if present):	TFA
Molecular mass (Av)	1229.341
Amount	10.0mg (1 x 9.0mg, 1 x 1.0mg)
Batch no	01
Purity (Determined by HPLC - see enc)	>90%
Physical properties	Lyophilised off-white powdered solid
Storage conditions	Store lyophilised at ≤ -20˚C

b

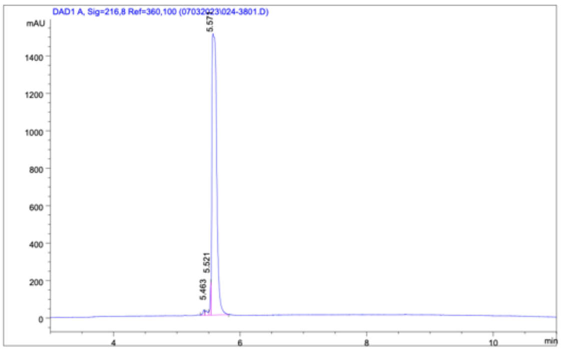

#	Meas. Ret.	Area	Area %
1	5.424	53.393	0.658
2	5.463	70.882	0.874
3	5.521	158.318	1.951
4	5.571	7831.374	96.517

c

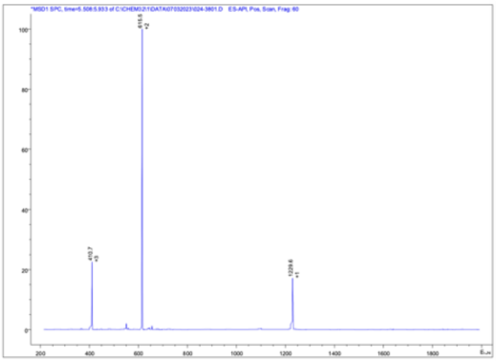

Purity and identification of peptides synthesised by Peptide Synthetics – Ac-ERWGHDFIK
a Peptide synthetics report summary. **b** HPLC trace. **c** Mass Spectrometry trace.

ERWGHDFIK-NH_2

a

University of St Andrews
Customer report: 63649

Date : 3rd March 2022

Compound code	
Sequence	ERWGHDFIK-NH2
N-terminus	Amine
C-terminus	Amide
Other modifications	None
Other/comments	
Counter Ion (if present):	TFA
Molecular mass (Av)	1186.319
Amount	5.0mg
Batch no	01
Purity (Determined by HPLC - see enc)	>90%
Physical properties	Lyophilised off-white powdered solid
Storage conditions	Store lyophilised at ≤ -20˚C

b

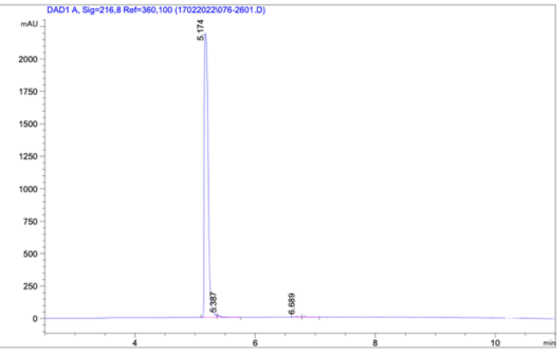

#	Meas. Ret.	Area	Area %
1	5.174	9452.316	98.918
2	5.387	64.951	0.680
3	6.689	17.973	0.188
4	6.817	20.498	0.215

c

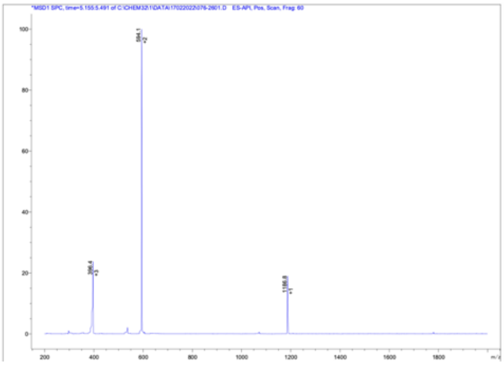

Purity and identification of peptides synthesised by Peptide Synthetics – ERWGHDFIK-NH2
a Peptide synthetics report summary. **b** HPLC trace. **c** Mass Spectrometry trace.

ERLGHDFIK

a

University of St Andrews
Customer report: 63420

Date :26th January 2022

Compound code	
Sequence	ERLGHDFIK
N-terminus	Amine
C-terminus	Acid
Other modifications	None
Other/comments	
Counter Ion (if present):	TFA
Molecular mass (Av)	1114.251
Amount	5.0mg
Batch no	01
Purity (Determined by HPLC - see enc)	>90%
Physical properties	Lyophilised off-white powdered solid
Storage conditions	Store lyophilised at ≤ -20˚C

b

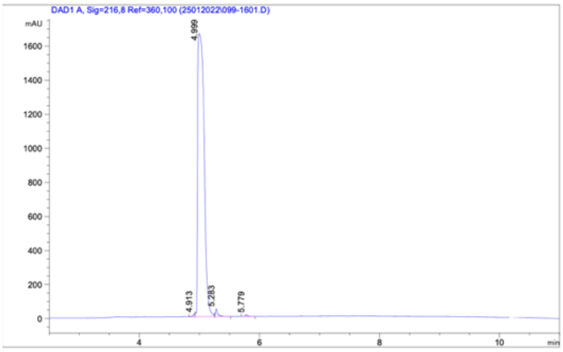

#	Meas. Ret.	Area	Area %
1	4.913	29.345	0.232
2	4.999	12474.088	98.671
3	5.283	115.987	0.917
4	5.779	22.651	0.179

c

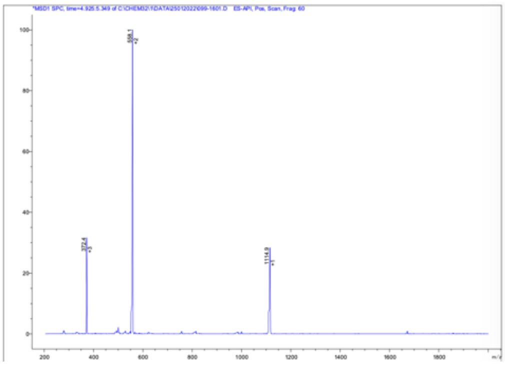

Purity and identification of peptides synthesised by Peptide Synthetics – ERLGHDFIK
a Peptide synthetics report summary. **b** HPLC trace. **c** Mass Spectrometry trace.

Supplementary Tables

Supplementary Table 1. Crystallographic Data Table

	**PaAP**	**PaAP_T**	**PaAP_T$_{(ERWGHDFIK)}$**	**PaAP_T$_{E340A}$**	**PaAP_T$_{E340A-trunc}$**
Accession code	8AC7	8AC9	8ACK	8ACR	8ACG
Resolution (Å)	85.95-1.4 (1.42-1.4)	66.73-2.35 (2.39-2.35)	64.46-1.78 (1.82-1.78)	52.75-2.10 (2.14-2.10)	61.13-2.84 (2.89-2.84)
Space group	P 1 21 1	P 1 21 1	P 1 21 1	P 2 21 21	P 3
Cell Dimensions a, b, c (Å)	62.95, 85.95, 98.22	77.00, 52.50, 114.38	63.29, 85.66, 98.10	46.02, 90.64, 129.72	186.74, 186.74, 84.73
α, β, γ (°)	90, 93.56, 90	90, 95.76, 90	90, 93.85, 90	90, 90, 90	90, 90, 120
Total reflections	1375192 (45920)	256358 (8870)	566021 (28343)	428078 (16810)	406620 (14467)
Unique reflections	203540 (8909)	38179 (1795)	98441 (4868)	32511 (1523)	77841 (3761)
Multiplicity	6.8 (5.2)	6.7 (4.9)	5.7 (5.8)	13.2 (11.0)	5.2 (3.8)
Completeness (%)	99.4 (87.7)	99.6 (92.6)	99.1 (98.7)	99.9 (96.9)	99.8 (97.3)
Mean I/sigma(I)	20.6 (1.1)	19.2 (2.6)	5.5 (0.7)	21.7 (1.0)	15.2 (0.8)
R-merge	0.057 (0.660)	0.068 (0.417)	0.114 (2.604)	0.057 (0.527)	0.056 (1.390)
R-pim	0.023 (0.316)	0.028 (0.204)	0.052 (1.167)	0.016 (0.164)	0.027 (0.791)
CC1/2	0.998 (0.820)	0.998 (0.911)	0.997 (0.362)	0.999 (0.928)	0.993 (0.354)
R-free	0.1749 (0.3383)	0.2343 (0.3329)	0.2101 (0.3819)	0.2238 (0.3162)	0.2385 (0.3830)
R-work	0.1558 (0.2980)	0.1829 (0.2150)	0.1713 (0.3433)	0.1759 (0.2545)	0.1907 (0.3323)
Total non-hydrogen atoms	8648	7278	8042	3876	21300
Total macromolecule atoms	7269	7115	7058	3678	21270
Total ligand atoms	61	7	209	4	21
Total solvent atoms	1318	156	787	194	9
Protein molecules per ASU	2	2	2	1	6
Residues per protein	477	472	468	485	472
RMS(bonds) (Å)	0.005	0.0089	0.007	0.0075	0.0098
RMS(angles) (°)	0.77	1.33	0.82	1.14	1.09
Ramachandran favoured (%)	97.36	96.59	97.32	96.89	96.44
Ramachandran allowed (%)	2.64	3.41	2.58	3.11	3.49
Ramachandran outliers (%)	0.00	0.00	0.11	0.00	0.07
Average B-factor (Å^2)	21.00	56.80	36.0	55.35	104.68

*Values in parentheses are for the high-resolution shell

**R-value test set size = 5%

Supplementary Table 2. Crystallisation conditions

Crystal	Condition
PaAP	0.1 M sodium acetate pH 4.5, 0.2 M zinc acetate, 10 % PEG3000, 20 % 2-propanol
PaAP_T	0.1 M HEPES pH 7.5, 70 % v/v MPD
PaaP_T$_{(ERWGHDFIK)}$	0.2 M zinc acetate, 0.1 M sodium cacodylate pH6.4, 11.7% 2-propanol
PaaP_T$_{E340A}$	0.1 M MMT (DL-Malic acid, MES monohydrate, Tris) pH 6.0, 25 % w/v PEG 1500
PaaP_T$_{E340A\text{-}trunc}$	0.1 M magnesium acetate tetrahydrate, 0.1 M potassium chloride, 0.1 M MES pH 6.2, 12 % v/v PEG Smear High*

* 50% PEG smear High = 16.7% w/v PEG 8000, 16.7% w/v PEG 10000, 16.7% w/v PEG 6000

Supplementary Table 3. Primers

		**Primers (5' – 3')**
**Strain Construction**		
**ΔPaAP**		
PA14_26020_del1		tgggtaacgccagggttttcccagtcacgacgttgtaaaaCGATGTCGCCGGGCGCGA
PA14_26020_del2		CCGGCGCAGGGTAGTTGCGGGAGACTCCGTTCCTTGTGAG
PA14_26020_del3		CTCACAAGGAACGGAGTCTCCCGCAACTACCCTGCGCCGG
PA14_26020_del4		gtgagcggataacaatttcacacaggaaacagctatgacGGGTACCAACCTGGAGAGC
**ΔPaAP_PaAPoex**		
PA14_26020_Pbad	F	actgtttctccatacccgtttttttgggctagcgaattcgAAGGAACGGAGTCTCATGAG
PA14_26020_Pbad	R	tctgttttatcagaccgcttctgcgttctgatttaaaaaTTACTTGATGAAGTCGTGACC
**Sequencing**		
PA14_26020_chkF	F	TCAGGAATTACGCCAGGACA
PA14_26020_chkR	R	CTGGAAGCGGTGGAGTGAT

**Recombinant Expression Constructs**		
PaAP	F	AAAATTTGTATTTCCAGGGGTCAGAAGCACAACAG<br>TTCACCG
	R	ATGCTAGGGGGAAGCTTTCATTTAATAAAGTCATG<br>ACCCCAGCGTTC
PaAP_T	F	CGTGGATCTCAAATGCAAAAGTCAGCCAGCC
	R	TGGAACTAGTTTCTGGCCTGCGGCAGC
PaaP_T_{E340A}	F	TGGGGTGCTGCAGAGGCGGGT
	R	CCAGGCGAAGCGTACCTTG
PaaP_$T_{E340A\text{-}trunc}$	F	AGTTCCACGTTGATCTCAAATGCAAAAGTC
	R	AGTTTCTGGCCTGCGGCA
PaaP_T_{Y466F}	F	GATGAGTGTTTCCATAGCAAATGCG
	R	GTAAGCTTTACCGGCCGT
PaaP_T_{R189A}	F	ACTTATTCAGgcTGGGACGTGTAACTTTGAGCAGAAAG
	R	GCAATGGATCCGGCAGGG

F = forward primer, R = reverse primer

Supplementary Table 4. Strains

	Strain	Description	source
**E coli**			
MB115	DH5a + pMQ30_pepB	suicide vector marked with GmR with counter-selectable SacB marker for markerless clean deletion of pepB	This study
MB117	DH5a + pUCTn7T-miniTn7T-GmR-PBAD-pepB	suicide vector marked with ampR, with Tn7site-flanked GmR resistance to be transferred into attB site along with arabinose-inducible pepB	This study
MB010	HB101 + pRK2013	helper strain carrying conjugation machinery, marked by KanR	ref: 10.1038/nprot.2006.24
MB011	SM10 + pTNS1	helper strain carrying Tn7 transposase marked by AmpR	ref: 10.1038/nprot.2006.24
**P aeruginosa**			
MB001	UCBPP-PA14	Wild Type, UCBPP-PA14	Kind gift from Dianne Newman lab; can cite: Mathee K. Forensic investigation into the origin of Pseudomonas aeruginosa PA14 – old but not lost. J Med Microbiol. 2018 Aug;67(8): 1019–21.
MB116	PA14 Δ*pepB*	UCBPP-PA14 with pepB locus precisely deleted	This study
MB118	PA14 Δ*pepB* attB::PBAD-pepB	UCBPP-PA14 with pepB locus precisely deleted and arabinose-inducible pepB construct inserted into attB (Tn7) locus immediately downstream of glmS gene	This study

Supplementary Table 5. Fitted data

	**Fit of first exponential - 200 mins**									
	**Peptide**	**Y0**	**error**	***k*₁**	**error**	**Equation fitted**	***k*₂**	**Error***	**Rate of linear phase**	**error**
P0	ERLGHDFIK	246090.55	6174.63	0.209	0.015	Single Exponential				
P-1	RLGHDFIK	16377.31	641.58	0.231	0.026	Double Exponential	0.0093	large		
P-2	LGHDFIK	11712.00	815.40	0.038	0.024	Single Exponential				
P-3	GHDFIK	434563.77	21401.75	0.031	0.006	Single Exponential				
P-4	HDFIK	86108.50	8836.07	0.084	0.005	Double Exponential	-0.0010	large		
P-5	DFIK	56122.63	4096.76	0.008	0.002	Single Exponential				
P-6	FIK	11261.04	873.27	0.014	0.007	Single Exponential+linear			67.19	2.47
P-7	IK	62000.00	3766.00	0.046	0.022	Double Exponential	0.0070	large		
	**Peptide**	**Y0**		***k*₁**	**error**		***k*₂**		**Half life second phase**	**error**
P0	ERWGHDFIK	425494.67	9942.62	0.004	0.001	Single Exponential				
P-1	RWGHDFIK	34429.00	3127.00	0.012		Double Exponential	0.0088	large	78.96	
P-2	WGHDFIK	42368.00	10936.00	0.009		Double Exponential	2.37E-06	large	292035.408	

P-3	GHDFIK	13419.10		0.000		10min lag+Single Exponential				
P-4	HDFIK	9876.91	3846.06	0.043	0.063	Single Exponential+line ar			245.80	5.11
P-5	DFIK	15546.40	2302.31	0.050		Single Exponential+line ar			47.32	2.86
	**Peptide**	**Y0**		**k_1**	**error**		**k2**			
P0	ERWGHDFIK-NH2	741171.00	14740.00	0.019	0.002	Single Exponential				
P-1	RWGHDFIK-NH2	172558.00	4579.00	0.021	0.002	Single Exponential				
P-2	WGHDFIK-NH2	426843.00	21060.00	0.027		Double Exponential	0.026	large		
P-3	GHDFIK-NH2	~ 4.930e-032	22965422698.00	0.019		Double Exponential	0.018	large		
P-4	HDFIK-NH2	894923.00	14900.00	0.018	0.003	10min lag+Single Exponential				
P-5	DFIK-NH2	139707.31	5643.89	0.017	0.003	30min lag+Single Exponential				
P-6	FIK-NH2	19642.00	3451.00	0.031	0.008	30min lag+Single Exponential				

P-7	IK-NH2	33955.00	5482.00	0.040	0.013	30min lag+Single Exponential				
	**Peptide**	**Y0**		**k_1**	**error**				**Rate of linear phase**	**error**
P0	HCATIPAFDG	457167.71	8861.48	0.031	0.012	Single Exponential				
P-1	CATIPAFDG	28031.01	836.33	0.012	0.005	Single Exponential				
P-2	ATIPAFDG	61372.94	1221.86	0.024	0.021	Single Exponential+linear			45.33	32.69
P-3	TIPAFDG	36776.42	527.02	0.013	0.001	Single Exponential				
P-4	IPAFDG	62156.79	894.91	0.005	0.001	Single Exponential				
P-5	PAFDG	745074.13	13397.88	0.043	0.021	Single Exponential+linear			545.8236	144.3407
P-6	AFDG	29680.84	542.87	0.015	0.008	Single Exponential				
P-7	FDG	58811.36	1235.24	0.016	0.004	Single Exponential				
P-8	DG	76528.35	1098.58	0.011	0.008	Single Exponential				
	**Peptide**	**Y0**		**k_1**	**error**				**Rate of linear phase**	**error**
P0	RWGHDFIK	1919994.64	136884.88	0.033	0.010	Single Exponential				
P-1	WGHDFIK	2812475.27	83146.92	0.006	0.001	Single Exponential				

P-2	GHDFIK					NA				
P-3	HDFIK	85632.87	80614.28	0.025	0.007	Single Exponential				
P-4	DFIK	473244.33	13644.31	0.000	0.000	Single Exponential				
P-5	FIK					NA				
P6	IK	122456.97	14481.84	0.030	0.034	Single Exponential+linear			276.60	46.44
	**Peptide**	**Y0**		**k_1**	**error**					
P0	KA-AMC	2050321.16	8684685264496900.00	0.089		Single Exponential				
P-1	A-AMC	0.00	676772.21	0.304	0.188	Double Exponential				
P-2	AMC	0.00	676772.21	0.304	0.188	Single Exponential				
	**Peptide**	**Y0**		**k_1**	**error**		**k_2**	**error**		
P0	KWLGYL	603459.76	20040.62	0.075	0.007	Single Exponential				
P-1	WLGYL	536088.38	21750.97	0.036	0.283	Double Exponential	0.035238	large		
P-2	LGYL					NA				
P0	KWLGYL-R189A	731234.61	19069.51	0.041	0.004	Single Exponential				
P-1	WLGYL-R189A	390399.44	24718.77	0.058	0.015	Double Exponential	0.008349	0.0016		
P-2	LGYL-R189A	100929.97	17078.11	0.014	0.306	Double Exponential	0.012244	large		

* errors labelled as “large” on k_2 as second exponential curve has no plateau – not to be interpreted quantitatively, rather as presence of a second phase.

References

(1) Desmarais, W.; Bienvenue, D. L.; Bzymek, K. P.; Petsko, G. A.; Ringe, D.; Holz, R. C. The high-resolution structures of the neutral and the low pH crystals of aminopeptidase from Aeromonas proteolytica. *Journal of Biological Inorganic Chemistry* **2006**, *11* (4), 398-408. DOI: 10.1007/s00775-006-0093-x. Fasciglione, G. F.; Marini, S.; D'Alessio, S.; Politi, V.; Coletta, M. pH- and Temperature-Dependence of Functional Modulation in Metalloproteinases. A Comparison between Neutrophil Collagenase and Gelatinases A and B. *Biophysical Journal* **2000**, *79* (4), 2138-2149. DOI: https://doi.org/10.1016/S0006-3495(00)76461-7. Gilboa, R.; Spungin-Bialik, A.; Wohlfahrt, G.; Schomburg, D.; Blumberg, S.; Shoham, G. Interactions of Streptomyces griseus aminopeptidase with amino acid reaction products and their implications toward a catalytic mechanism. *Proteins-Structure Function and Genetics* **2001**, *44* (4), 490-504. DOI: 10.1002/prot.1115.
