# Supplementary material for: An anti-biofilm cyclic peptide targets a secreted aminopeptidase from P. aeruginosa
Source: Nat Chem Biol. 2023 Jun 29;19(9):1158–66. doi: 10.1038/s41589-023-01373-8 (PMC10449631; doi:10.1038/s41589-023-01373-8)
Supplement: Supplementary file 2 — Reporting Summary [file 41589_2023_1373_MOESM2_ESM.pdf]

nature portfolio

Corresponding author(s): Clarissa Melo Czekster

Last updated by author(s): 2023/30/04

Reporting Summary

Nature Portfolio wishes to improve the reproducibility of the work that we publish. This form provides structure for consistency and transparency in reporting. For further information on Nature Portfolio policies, see our Editorial Policies and the Editorial Policy Checklist.

Statistics

For all statistical analyses, confirm that the following items are present in the figure legend, table legend, main text, or Methods section.

n/a	Confirmed	
☐	☒	The exact sample size (*n*) for each experimental group/condition, given as a discrete number and unit of measurement
☐	☒	A statement on whether measurements were taken from distinct samples or whether the same sample was measured repeatedly
☐	☒	The statistical test(s) used AND whether they are one- or two-sided<br>*Only common tests should be described solely by name; describe more complex techniques in the Methods section.*
☐	☒	A description of all covariates tested
☐	☒	A description of any assumptions or corrections, such as tests of normality and adjustment for multiple comparisons
☐	☒	A full description of the statistical parameters including central tendency (e.g. means) or other basic estimates (e.g. regression coefficient) AND variation (e.g. standard deviation) or associated estimates of uncertainty (e.g. confidence intervals)
☐	☒	For null hypothesis testing, the test statistic (e.g. *F*, *t*, *r*) with confidence intervals, effect sizes, degrees of freedom and *P* value noted<br>*Give P values as exact values whenever suitable.*
☒	☐	For Bayesian analysis, information on the choice of priors and Markov chain Monte Carlo settings
☒	☐	For hierarchical and complex designs, identification of the appropriate level for tests and full reporting of outcomes
☒	☐	Estimates of effect sizes (e.g. Cohen's *d*, Pearson's *r*), indicating how they were calculated

Our web collection on statistics for biologists contains articles on many of the points above.

Software and code

Policy information about availability of computer code

Data collection	Crystallograhic data were collected using Diamond Light source collection, other data were collected in house
Data analysis	Graphpad prism, ChimeraX, CCP4 suite, Phenix, ImageJ, BiofilmQ. Versions have been provided in the manuscript

For manuscripts utilizing custom algorithms or software that are central to the research but not yet described in published literature, software must be made available to editors and reviewers. We strongly encourage code deposition in a community repository (e.g. GitHub). See the Nature Portfolio guidelines for submitting code & software for further information.

Data

Policy information about availability of data

All manuscripts must include a data availability statement. This statement should provide the following information, where applicable:

- Accession codes, unique identifiers, or web links for publicly available datasets
- A description of any restrictions on data availability
- For clinical datasets or third party data, please ensure that the statement adheres to our policy

Data availability
Structural data were deposited in the PDB and are available under accession numbers 8ACR, 8ACK, 8AC7, 8AC9, 8ACG. Search components for molecular replacement comprised an ensemble of 3 PDBs previously deposited by others (6HC6, 5IB9 and 1TKJ). All other data are contained in the main manuscript, and supplementary information, raw data available for download on Figshare. All materials and reagents are available from the corresponding authors.

Human research participants

Policy information about studies involving human research participants and Sex and Gender in Research.

Reporting on sex and gender	NA
Population characteristics	NA
Recruitment	NA
Ethics oversight	NA

Note that full information on the approval of the study protocol must also be provided in the manuscript.

Field-specific reporting

Please select the one below that is the best fit for your research. If you are not sure, read the appropriate sections before making your selection.

☒ Life sciences ☐ Behavioural & social sciences ☐ Ecological, evolutionary & environmental sciences

For a reference copy of the document with all sections, see nature.com/documents/nr-reporting-summary-flat.pdf

Life sciences study design

All studies must disclose on these points even when the disclosure is negative.

Sample size	For enzymatic assays, all done in triplicate. This is the standard in the field (enzymology) for kinetic assays, and determined to be adequate by individual inspection of each individual replicate, with the observation they did not deviate significantly (slope of individual curves within 10% of one another).<br>Biofilm assays were done as 4 biological replicates, and 10 randomly chosen spots were imaged and used for quantification. Sample size was chosen due to spacing on microscope slide and plate, and so samples and control experiments would be carried out at the same time.
Data exclusions	No data were excluded
Replication	Biofilm imaging was performed as 4 independent biological replicates, and a Kruskal-Wallis test was performed and there was no significance between biological replicates. A normality test was performed and samples were analysed with a nonparametric test if required. We also confirmed that the WT grew the same in 2 independent experiments (WT vs PaAP and Wt +/- peptide). All other data (kinetics, inhibition) was performed in triplicate, and data was globally fitted whenever possible. Kinetics and inhibition data were repeated in at least one independent experiment, showing data were reproducible.
Randomization	Randomization was applied for biofilm growth images so data analysis could be carried out. Other randomization was not applicable (for kinetic assays for example).
Blinding	Because cells and enzymatic assays needed to treated with an inhibitor, blinding was not possible as it was obvious which sample contained compound.

Reporting for specific materials, systems and methods

We require information from authors about some types of materials, experimental systems and methods used in many studies. Here, indicate whether each material, system or method listed is relevant to your study. If you are not sure if a list item applies to your research, read the appropriate section before selecting a response.

Materials & experimental systems

n/a	Involved in the study	
☒	☐	Antibodies
☒	☐	Eukaryotic cell lines
☒	☐	Palaeontology and archaeology
☒	☐	Animals and other organisms
☒	☐	Clinical data
☒	☐	Dual use research of concern

Methods

n/a	Involved in the study	
☒	☐	ChIP-seq
☒	☐	Flow cytometry
☒	☐	MRI-based neuroimaging
